# Likelihood Ratio Test for Excess Homozygosity at Marker Loci on X Chromosome

**DOI:** 10.1371/journal.pone.0145032

**Published:** 2015-12-15

**Authors:** Xiao-Ping You, Qi-Lei Zou, Jian-Long Li, Ji-Yuan Zhou

**Affiliations:** State Key Laboratory of Organ Failure Research and Guangdong Provincial Key Laboratory of Tropical Research, School of Public Health and Tropical Medicine, Southern Medical University, Guangzhou, Guangdong, China; NIH - National Institute of Environmental Health Sciences, UNITED STATES

## Abstract

The assumption of Hardy-Weinberg equilibrium (HWE) is generally required for association analysis using case-control design on autosomes; otherwise, the size may be inflated. There has been an increasing interest of exploring the association between diseases and markers on X chromosome and the effect of the departure from HWE on association analysis on X chromosome. Note that there are two hypotheses of interest regarding the X chromosome: (i) the frequencies of the same allele at a locus in males and females are equal and (ii) the inbreeding coefficient in females is zero (without excess homozygosity). Thus, excess homozygosity and significantly different minor allele frequencies between males and females are used to filter X-linked variants. There are two existing methods to test for (i) and (ii), respectively. However, their size and powers have not been studied yet. Further, there is no existing method to simultaneously detect both hypotheses till now. Therefore, in this article, we propose a novel likelihood ratio test for both (i) and (ii) on X chromosome. To further investigate the underlying reason why the null hypothesis is statistically rejected, we also develop two likelihood ratio tests for detecting (i) and (ii), respectively. Moreover, we explore the effect of population stratification on the proposed tests. From our simulation study, the size of the test for (i) is close to the nominal significance level. However, the size of the excess homozygosity test and the test for both (i) and (ii) is conservative. So, we propose parametric bootstrap techniques to evaluate their validity and performance. Simulation results show that the proposed methods with bootstrap techniques control the size well under the respective null hypothesis. Power comparison demonstrates that the methods with bootstrap techniques are more powerful than those without bootstrap procedure and the existing methods. The application of the proposed methods to a rheumatoid arthritis dataset indicates their utility.

## Introduction

Association analysis is a useful tool to map disease loci by using markers on autosomes based on family data and case-control data [1–9]. There has been an increasing interest of exploring the association between diseases and markers on X chromosome and the effect of the departure from Hardy-Weinberg equilibrium (HWE) on association analysis on X chromosome [10–17]. Note that there are two hypotheses of interest regarding the X chromosome: (i) the frequencies of the same allele at a locus in males and females are equal and (ii) the inbreeding coefficient in females is zero (without excess homozygosity) in X-specific quality control [18, 19]. As such, excess homozygosity in females and significantly different minor allele frequencies between males and females are used to filter X-linked variants [20, 21]. The inbreeding coefficient is generally estimated by functions of excess homozygosity [22, 23], which may be caused by population substructure, consanguineous mating or factors like null alleles [24, 25]. Overall and Nichols developed an approach to distinguish population substructure and consanguinity by using multilocus genotype data [24]. On the other hand, Zheng et al. proposed two test statistics to test for the equality of the frequencies of the same allele in males and females and the zero inbreeding coefficient in females on X chromosome, respectively [14]. However, they only focused on association analysis on X chromosome and the type I error rates and powers of these two test statistics have not been studied yet. Further, there is no existing method to simultaneously detect both of the issues till now.

Therefore, in this article, we first combine two test statistics proposed in zheng et al. [14] and suggest *Z*_0_ to simultaneously test for (i) the equality of the frequencies of the same allele in males and females and (ii) the zero inbreeding coefficient on X chromosome based on the collected sample. For the purpose of improving the test power for both (i) and (ii), a novel likelihood ratio test on X chromosome is proposed. We write out the likelihood functions of the collected sample under the null hypothesis and alternative hypothesis at a single locus on X chromosome, respectively. Next, we obtain the maximum likelihood estimates (MLEs) of the unknown parameters by expectation-maximization (EM) algorithms [26] and construct the corresponding likelihood ratio test (LRT_0_) statistic to test for both (i) and (ii). If the null hypothesis is statistically rejected, we further conduct two hypothesis testing issues to find the underlying reasons why the null hypothesis is violated by proposing another two likelihood ratio tests LRT_1_ (for the equality of the frequencies of the same allele in males and females) and LRT_2_ (for excess homozygosity). Note that the size of LRT_0_ and LRT_2_ is conservative from our simulation study. As such, we use parametric bootstrap techniques to evaluate the validity and performance of LRT_0_ and LRT_2_, which are respectively denoted by LRT_0*b*_ and LRT_2*b*_. Moreover, we explore the effect of population stratification on the proposed tests. In addition, the root mean squared error (RMSE) and bias are used to assess the accuracy of the MLEs of the unknown parameters. Finally, the application of the proposed methods to a rheumatoid arthritis (RA) dataset indicates their utility.

## Materials and Methods

### Background and notations

Consider a biallelic marker locus on X chromosome with alleles *M*_1_ and *M*_2_. Let *p*_*m*_ and *p*_*f*_ be the frequencies of *M*_1_ in males and females, respectively. As such, the frequencies of *M*_2_ in males and females are *q*_*m*_ = 1 − *p*_*m*_ and *q*_*f*_ = 1 − *p*_*f*_, respectively. In females, let *ρ* be the inbreeding coefficient, which is generally nonnegative [27–29]. Thus, the frequencies of three genotypes *M*_1_ *M*_1_, *M*_1_ *M*_2_ and *M*_2_ *M*_2_ in females can be expressed as follows:

$$\begin{array}{c} P\left(M_{1}M_{1}\right)=p_{f}^{2}+\rho p_{f}q_{f},P\left(M_{1}M_{2}\right)=2\left(1-\rho \right)p_{f}q_{f},P\left(M_{2}M_{2}\right)=q_{f}^{2}+\rho p_{f}q_{f}. \end{array}$$

To this end, there is no excess homozygosity in females when *ρ* = 0; excess homozygosity exists when *ρ* > 0. Note that *p*_*m*_ ≠ *p*_*f*_ may be true on X chromosome. So, we construct the null hypothesis denoted by *H*_0_: *p*_*m*_ = *p*_*f*_ and *ρ* = 0 to test for both of the hypotheses (i) and (ii). If the null hypothesis is violated, we need to investigate which one of *p*_*m*_ ≠ *p*_*f*_ and *ρ* > 0 is true. As such, we have other two hypothesis testing issues with the null hypothesis being *H*_01_: *p*_*m*_ = *p*_*f*_ and *H*_02_: *ρ* = 0, respectively. It should be noted that X chromosome has the problem of X chromosome inactivation and dosage compensation [30], but we do not consider them in this section. The corresponding discussion can be found later (see the Discussion section).

Assume that *n*_1*m*_ and *n*_0*m*_ represent the numbers of males with alleles *M*_1_ and *M*_2_ in a collected sample, respectively; *n*_2*f*_, *n*_1*f*_ and *n*_0*f*_ denote the numbers of females with genotypes *M*_1_ *M*_1_, *M*_1_ *M*_2_ and *M*_2_ *M*_2_, respectively. Then, *N*_*m*_ = *n*_1*m*_ + *n*_0*m*_ and *N*_*f*_ = *n*_2*f*_ + *n*_1*f*_ + *n*_0*f*_ are respectively the numbers of males and females in the sample, and *N* = *N*_*m*_ + *N*_*f*_ is the sample size.

### Existing methods *Z*_1_ and *Z*_2_ for *H*_01_ (equality of the frequencies of the same allele in males and females) and *H*_02_ (zero inbreeding coefficient), respectively

Zheng et al. [14] proposed the test statistic

$$\begin{array}{c} Z_{1}=\frac{{\left({\hat{p}}_{m}-{\hat{p}}_{f}\right)}^{2}}{Var\left({\hat{p}}_{m}\right)+Var\left({\hat{p}}_{f}\right)} \end{array}$$

to test for *H*_01_: *p*_*m*_ = *p*_*f*_, where ${\hat{p}}_{m}=n_{1m}/N_{m}$ and ${\hat{p}}_{f}=\left(2n_{2f}+n_{1f}\right)/\left(2N_{f}\right)$ are the estimates of *p*_*m*_ and *p*_*f*_, $Var\left({\hat{p}}_{m}\right)={\hat{p}}_{m}\left(1-{\hat{p}}_{m}\right)/N_{m}$, and $Var\left({\hat{p}}_{f}\right)=\left[{\hat{p}}_{f}-2{\hat{p}}_{f}^{2}+\hat{P}\left(M_{1}M_{1}\right)\right]/\left(2N_{f}\right)$ are the estimates of the variances of ${\hat{p}}_{m}$ and ${\hat{p}}_{f}$ under *H*_01_, respectively, with $\hat{P}\left(M_{1}M_{1}\right)=n_{2f}/N_{f}$. Under *H*_01_, *Z*_1_ asymptotically follows the chi-square distribution with one degree of freedom when the sample size is large enough.

Weir and cockerham [31] introduced the disequilibrium coefficient in females $\Delta_{f}=P\left(M_{1}M_{1}\right)-p_{f}^{2}=\rho p_{f}q_{f}$. In other words, testing for Δ_*f*_ = 0 is equivalent to testing for *ρ* = 0. Hence, zheng et al. [14] further developed the following test statistic to test for *H*_02_: *ρ* = 0,

$$\begin{array}{c} Z_{2}=\frac{{\left[{\hat{\Delta }}_{f}+{\hat{p}}_{f}{\hat{q}}_{f}/\left(2N_{f}\right)\right]}^{2}}{Var({\hat{\Delta }}_{f})}=\frac{N_{f}{\left[{\hat{\Delta }}_{f}+{\hat{p}}_{f}{\hat{q}}_{f}/\left(2N_{f}\right)\right]}^{2}}{{{\hat{p}}_{f}}^{2}{{\hat{q}}_{f}}^{2}}, \end{array}$$

where ${\hat{\Delta }}_{f}=\hat{P}\left(M_{1}M_{1}\right)-{\hat{p}}_{f}^{2}$, ${\hat{q}}_{f}=1-{\hat{p}}_{f}$, $E\left({\hat{\Delta }}_{f}\right)=-p_{f}q_{f}/\left(2N_{f}\right)$ and $Var\left({\hat{\Delta }}_{f}\right)=p_{f}^{2}{q_{f}}^{2}/N_{f}$. Under *H*_02_, *Z*_2_ approximately follows the chi-square distribution with one degree of freedom when *N*_*f*_ is large enough. It should be noted that the test *Z*_2_ has nothing to do with male individuals and thus only needs female individuals.

### *Z*_0_ test for both hypotheses (i) and (ii) of interest regarding the X chromosome

Zheng et al. [14] showed that, under *H*_0_: *p*_*m*_ = *p*_*f*_ and *ρ* = 0, *Z*_1_ and *Z*_2_ are independent. However, they did not propose the corresponding test statistic for *H*_0_. As such, we suggest the test statistic

$$\begin{array}{c} Z_{0}=Z_{1}+Z_{2} \end{array}$$

to test for *H*_0_: *p*_*m*_ = *p*_*f*_ and *ρ* = 0. Under *H*_0_, *Z*_0_ asymptotically follows the chi-square distribution with the degrees of freedom being 2. Moreover, it should be noted that we can use

$$\begin{array}{c} {\hat{\rho }}_{z}=\frac{\hat{P}\left(M_{1}M_{1}\right)-{\hat{p}}_{f}^{2}}{{\hat{p}}_{f}{\hat{q}}_{f}} \end{array}$$

to estimate the inbreeding coefficient *ρ*.

### Likelihood ratio test for both hypotheses (i) and (ii) of interest regarding the X chromosome

To construct a likelihood ratio test (LRT) for *H*_0_: *p*_*m*_ = *p*_*f*_ and *ρ* = 0, we give the likelihood function of the sample as follows:

$$\begin{array}{ccc} L\left(\theta \right) & = & \left(\begin{array}{c} N_{m}\\ n_{1m} \end{array}\right)\left(\begin{array}{c} N_{f}\\ n_{2f},n_{1f},n_{0f} \end{array}\right)p_{m}^{n_{1m}}q_{m}^{n_{0m}}{\left(p_{f}^{2}+\rho p_{f}q_{f}\right)}^{n_{2f}}\\ & & \times {\left[2\left(1-\rho \right)p_{f}q_{f}\right]}^{n_{1f}}{\left(q_{f}^{2}+\rho p_{f}q_{f}\right)}^{n_{0f}}, \end{array}$$ (1)

where *θ* = (*p*_*m*_, *p*_*f*_, *ρ*). Firstly, we use the following EM algorithm to estimate the unknown parameters *p*_*m*_, *p*_*f*_ and *ρ* under the alternative hypothesis (*H*_1_: *p*_*m*_ ≠ *p*_*f*_ or *ρ* > 0). Suppose that *Y* = (*Y*_1_, *Y*_2_, *Y*_3_, *Y*_4_, *Y*_5_) = (*n*_1*m*_, *n*_0*m*_, *n*_2*f*_, *n*_1*f*_, *n*_0*f*_) denotes the observed data. (*Y*_1_, *Y*_2_, *Y*_3_, *Y*_4_, *Y*_5_) can be augmented by splitting the third cell into two cells *W*_1_ and *W*_2_, which are unobservable random variables such that *Y*_3_ = *W*_1_ + *W*_2_ for female homozygote *M*_1_ *M*_1_ and *W*_1_ and *W*_2_ follow the binomial distributions with success probabilities $p_{f}^{2}/\left(p_{f}^{2}+\rho p_{f}q_{f}\right)$ and $\rho p_{f}q_{f}/\left(p_{f}^{2}+\rho p_{f}q_{f}\right)$, respectively, and by splitting the fifth cell into two cells *W*_3_ and *W*_4_, where *Y*_5_ = *W*_3_ + *W*_4_ for female homozygote *M*_2_ *M*_2_ and *W*_3_ and *W*_4_ follow the binomial distributions with success probabilities $q_{f}^{2}/\left(q_{f}^{2}+\rho p_{f}q_{f}\right)$ and $\rho p_{f}q_{f}/\left(q_{f}^{2}+\rho p_{f}q_{f}\right)$, respectively. Thus, the likelihood function of complete data (*n*_1*m*_, *n*_0*m*_, *w*_1_, *w*_2_, *n*_1*f*_, *w*_3_, *w*_4_) is:

$$\begin{array}{ccc} L_{c}\left(\theta \right) & \propto & p_{m}^{n_{1m}}q_{m}^{n_{0m}}p_{f}^{2w_{1}+w_{2}+n_{1f}+w_{4}}q_{f}^{w_{2}+n_{1f}+2w_{3}+w_{4}}\rho^{w_{2}+w_{4}}{\left(1-\rho \right)}^{n_{1f}}, \end{array}$$

where the normalizing constant is omitted for brevity.

At the E-step, the *Q* function at iteration (*k* + 1) is constructed as

$$\begin{array}{ccc} Q(\theta |\theta^{\left(k\right)}) & = & n_{1m}\ln p_{m}+n_{0m}\ln q_{m}\\ & & +\left[2E_{\theta^{\left(k\right)}}\left(w_{1}|n_{2f}\right)+E_{\theta^{\left(k\right)}}\left(w_{2}|n_{2f}\right)+n_{1f}+E_{\theta^{\left(k\right)}}\left(w_{4}|n_{0f}\right)\right]\ln p_{f}\\ & & +\left[E_{\theta^{\left(k\right)}}\left(w_{2}|n_{2f}\right)+n_{1f}+2E_{\theta^{\left(k\right)}}\left(w_{3}|n_{0f}\right)+E_{\theta^{\left(k\right)}}\left(w_{4}|n_{0f}\right)\right]\ln\left(1-p_{f}\right)\\ & & +\left[E_{\theta^{\left(k\right)}}\left(w_{2}|n_{2f}\right)+E_{\theta^{\left(k\right)}}\left(w_{4}|n_{0f}\right)\right]\ln\rho +n_{1f}\ln\left(1-\rho \right), \end{array}$$

where *θ* ^(*k*)^ is the estimate of *θ* at iteration *k*.

At the M-step, the estimated value *θ* ^(*k*+1)^ of *θ* at iteration (*k* + 1) can be obtained by maximizing the *Q* function with respect to *θ*. Therefore, the MLEs of *p*_*m*_, *p*_*f*_ and *ρ* at iteration (*k* + 1) are respectively

$$\begin{array}{ccc} {\hat{p}}_{m} & = & \frac{n_{1m}}{N_{m}},\\ {\hat{p}}_{f1}^{\left(k+1\right)} & = & \frac{E_{\theta^{\left(k\right)}}\left(2w_{1}+w_{2}|n_{2f}\right)+n_{1f}+E_{\theta^{\left(k\right)}}\left(w_{4}|n_{0f}\right)}{2N_{f}},\\ {\hat{\rho }}_{1}^{\left(k+1\right)} & = & \frac{E_{\theta^{\left(k\right)}}\left(w_{2}|n_{2f}\right)+E_{\theta^{\left(k\right)}}\left(w_{4}|n_{0f}\right)}{E_{\theta^{\left(k\right)}}\left(w_{2}|n_{2f}\right)+E_{\theta^{\left(k\right)}}\left(w_{4}|n_{0f}\right)+n_{1f}}. \end{array}$$

Note that the MLE of *p*_*m*_ is the same for all the iterations, which is also the same as zheng et al. [14]. In the above expressions,

$$\begin{array}{ccc} E_{\theta^{\left(k\right)}}\left(w_{1}|n_{2f}\right) & = & \frac{n_{2f}{\left({\hat{p}}_{f1}^{\left(k\right)}\right)}^{2}}{{\left({\hat{p}}_{f1}^{\left(k\right)}\right)}^{2}+{\hat{\rho }}_{1}^{\left(k\right)}{\hat{p}}_{f1}^{\left(k\right)}{\hat{q}}_{f1}^{\left(k\right)}}, \end{array}$$ (2)

$$\begin{array}{ccc} E_{\theta^{\left(k\right)}}\left(w_{2}|n_{2f}\right) & = & \frac{n_{2f}{\hat{\rho }}_{1}^{\left(k\right)}{\hat{p}}_{f1}^{\left(k\right)}{\hat{q}}_{f1}^{\left(k\right)}}{{\left({\hat{p}}_{f1}^{\left(k\right)}\right)}^{2}+{\hat{\rho }}_{1}^{\left(k\right)}{\hat{p}}_{f1}^{\left(k\right)}{\hat{q}}_{f1}^{\left(k\right)}}, \end{array}$$ (3)

$$\begin{array}{ccc} E_{\theta^{\left(k\right)}}\left(w_{3}|n_{{}_{0f}}\right) & = & \frac{n_{{}_{0f}}{\left({\hat{q}}_{f1}^{\left(k\right)}\right)}^{2}}{{\left({\hat{q}}_{f1}^{\left(k\right)}\right)}^{2}+{\hat{\rho }}_{1}^{\left(k\right)}{\hat{p}}_{f1}^{\left(k\right)}{\hat{q}}_{f1}^{\left(k\right)}}, \end{array}$$ (4)

$$\begin{array}{ccc} E_{\theta^{\left(k\right)}}\left(w_{4}|n_{{}_{0f}}\right) & = & \frac{n_{0f}{\hat{\rho }}_{1}^{\left(k\right)}{\hat{p}}_{f1}^{\left(k\right)}{\hat{q}}_{f1}^{\left(k\right)}}{{\left({\hat{q}}_{f1}^{\left(k\right)}\right)}^{2}+{\hat{\rho }}_{1}^{\left(k\right)}{\hat{p}}_{f1}^{\left(k\right)}{\hat{q}}_{f1}^{\left(k\right)}}, \end{array}$$ (5)

where ${\hat{q}}_{f1}^{\left(k\right)}=1-{\hat{p}}_{f1}^{\left(k\right)}$. Given the initial value *θ* ^(0)^ of *θ*, the above-mentioned two steps continue until the convergence criterion is satisfied. For example, the absolute differences between the estimates of the parameters at two consecutive iterations are all less than 10^−7^. The value of *θ* obtained at the last iteration is taken as the MLE ${\hat{\theta }}_{1}=\left({\hat{p}}_{m},{\hat{p}}_{f1},{\hat{\rho }}_{1}\right)$ of *θ* under *H*_1_.

Note that *p*_*m*_ = *p*_*f*_ and *ρ* = 0 under *H*_0_. Let *p* = *p*_*m*_ = *p*_*f*_, the pooled allele frequency of *M*_1_. Then, *L*(*θ*) in Eq (1) can be rewritten as

$$\begin{array}{c} L\left(\theta \right)\propto p^{n_{1m}+2n_{2f}+n_{1f}}{\left(1-p\right)}^{n_{0m}+n_{1f}+2n_{0f}}. \end{array}$$

Thus, the MLE of *p* under *H*_0_ is $\hat{p}=\left(n_{1m}+2n_{2f}+n_{1f}\right)/\left(N_{m}+2N_{f}\right)$, the estimated pooled allele frequency of *M*_1_. Let ${\hat{\theta }}_{0}=\left(\hat{p},\hat{p},0\right)$. Then, we can construct the following LRT to test for *H*_0_

$$\begin{array}{c} {\text{LRT}}_{0}=2\ln\frac{L({\hat{\theta }}_{1})}{L({\hat{\theta }}_{0})}, \end{array}$$ (6)

which asymptotically follows a chi-square distribution with the degrees of freedom being 2 when the null hypothesis holds.

### Likelihood ratio test for equality of frequencies of the same allele in males and females

Once the null hypothesis (*H*_0_: *p*_*m*_ = *p*_*f*_ and *ρ* = 0) is rejected based on the result of Eq (6), we further need to consider the following two tests *H*_01_: *p*_*m*_ = *p*_*f*_ and *H*_02_: *ρ* = 0. Note that under the null hypothesis *H*_01_: *p*_*m*_ = *p*_*f*_ = *p*, *ρ* may not be zero and we need to estimate it. Let *ϕ* = (*p*, *ρ*) and *q* = 1 − *p*. Thus, the corresponding likelihood function of complete data is

$$\begin{array}{ccc} L_{c_{1}}\left(\phi \right) & \propto & p^{n_{1m}+2w_{1}+w_{2}+n_{1f}+w_{4}}{\left(1-p\right)}^{n_{0m}+w_{2}+n_{1f}+2w_{3}+w_{4}}\rho^{w_{2}+w_{4}}{\left(1-\rho \right)}^{n_{1f}}. \end{array}$$

We use the following EM algorithm to estimate *ϕ* under *H*_01_. The corresponding formulas at iteration (*k* + 1) are as follows

$$\begin{array}{ccc} {\hat{p}}_{01}^{\left(k+1\right)} & = & \frac{E_{\phi^{\left(k\right)}}\left(2w_{1}+w_{2}|n_{2f}\right)+E_{\phi^{\left(k\right)}}\left(w_{4}|n_{0f}\right)+n_{1f}+n_{1m}}{N_{m}+2N_{f}},\\ {\hat{\rho }}_{01}^{\left(k+1\right)} & = & \frac{E_{\phi^{\left(k\right)}}\left(w_{2}|n_{2f}\right)+E_{\phi^{\left(k\right)}}\left(w_{4}|n_{0f}\right)}{E_{\phi^{\left(k\right)}}\left(w_{2}|n_{2f}\right)+E_{\phi^{\left(k\right)}}\left(w_{4}|n_{0f}\right)+n_{1f}}, \end{array}$$

where ${\hat{p}}_{01}^{\left(k+1\right)}$ and ${\hat{\rho }}_{01}^{\left(k+1\right)}$ are respectively the MLEs of *p* and *ρ* at iteration (*k* + 1), and $\phi^{\left(k\right)}=\left({\hat{p}}_{01}^{\left(k\right)},{\hat{\rho }}_{01}^{\left(k\right)}\right)$. *E*_*ϕ*^(*k*)^_(*w*_1_|*n*_2*f*_), *E*_*ϕ*^(*k*)^_(*w*_2_|*n*_2*f*_), *E*_*ϕ*^(*k*)^_(*w*_3_|*n*_0*f*_) and *E*_*ϕ*^(*k*)^_(*w*_4_|*n*_0*f*_) in the above expressions are similar to *E*_*θ*^(*k*)^_(*w*_1_|*n*_2*f*_), *E*_*θ*^(*k*)^_(*w*_2_|*n*_2*f*_), *E*_*θ*^(*k*)^_(*w*_3_|*n*_0*f*_) and *E*_*θ*^(*k*)^_(*w*_4_|*n*_0*f*_) in Eqs (2)–(5), just replacing ${\hat{p}}_{f1}^{\left(k\right)}$, ${\hat{q}}_{f1}^{\left(k\right)}$ and ${\hat{\rho }}_{1}^{\left(k\right)}$ in Eqs (2)–(5) by ${\hat{p}}_{01}^{\left(k\right)}$, ${\hat{q}}_{01}^{\left(k\right)}$ and ${\hat{\rho }}_{01}^{\left(k\right)}$, respectively. Let ${\hat{\theta }}_{01}=\left({\hat{p}}_{01},{\hat{p}}_{01},{\hat{\rho }}_{01}\right)$. Then, we propose the following test statistic LRT_1_ to test for the null hypothesis *H*_01_: *p*_*m*_ = *p*_*f*_,

$$\begin{array}{c} {\text{LRT}}_{1}=2\ln\frac{L({\hat{\theta }}_{1})}{L({\hat{\theta }}_{01})}, \end{array}$$ (7)

which approximately follows a chi-square distribution with the degree of freedom being 1 under *H*_01_.

### Likelihood ratio test for inbreeding coefficient being zero

Note that under the null hypothesis *H*_02_ : *ρ* = 0, *p*_*m*_ and *p*_*f*_ may be different from each other and we need to estimate them separately. Let *ψ* = (*p*_*m*_, *p*_*f*_) and *L*(*θ*) in Eq (1) can be rewritten as

$$\begin{array}{c} L_{2}\left(\psi \right)\propto p_{m}^{n_{1m}}q_{m}^{n_{0m}}p_{f}^{2n_{2f}+n_{1f}}q_{f}^{n_{1f}+2n_{0f}}. \end{array}$$

Then, the MLEs of *p*_*m*_ and *p*_*f*_ are ${\hat{p}}_{m}=n_{1m}/N_{m}$ and ${\hat{p}}_{f}=\left(2n_{2f}+n_{1f}\right)/\left(2N_{f}\right)$, respectively, which are the same as zheng et al. [14]. Let ${\hat{\theta }}_{02}=\left({\hat{p}}_{m},{\hat{p}}_{f},0\right)$. As such, we develop the following test statistic to test for *H*_02_ : *ρ* = 0

$$\begin{array}{c} {\text{LRT}}_{2}=2\ln\frac{L({\hat{\theta }}_{1})}{L({\hat{\theta }}_{02})}, \end{array}$$ (8)

which asymptotically follows a chi-square distribution with the degree of freedom being 1 under *H*_02_. Just like the *Z*_2_ test statistic, LRT_2_ only uses female individuals in the sample because the terms based on male individuals in the numerator and the denominator of the fraction are the same, which can be reduced.

### Likelihood ratio tests via parametric bootstrap for *H*_0_ and *H*_02_

It should be noted from our simulation results (see the Results section) that the simulated type I error rates of LRT_0_ and LRT_02_ respectively for *H*_0_ and *H*_02_ are too conservative. On the other hand, several studies showed that the likelihood ratio tests may typically not follow a chi-square distribution asymptotically [31, 32], and hence their exact distributions can be obtained by Monte Carlo simulation [33]. Accordingly, we make use of parametric bootstrap techniques to evaluate the size and power of these two methods. For convenience, we denote these methods via parametric bootstrap by LRT_0*b*_ and LRT_2*b*_, respectively. We begin by describing the implementation steps for LRT_0*b*_ as follows:

1. For a collected sample of size *N* with *N*_*m*_ males and *N*_*f*_ females, calculate the value of LRT_0_;
2. Compute the estimated pooled allele frequency $\hat{p}$ based on the sample as follows: $\hat{p}=\left(n_{1m}+2n_{2f}+n_{1f}\right)/\left(N_{m}+2N_{f}\right)$;
3. Based on $\hat{p}$, calculate the frequencies of three genotypes *M*_1_ *M*_1_, *M*_1_ *M*_2_ and *M*_2_ *M*_2_ in females under *H*_0_ in the following: ${\hat{p}}^{2}$, $2\hat{p}\hat{q}$ and ${\hat{q}}^{2}$, respectively, where $\hat{q}=1-\hat{p}$;
4. According to $\hat{p}$ and $\hat{q}$, regenerate the alleles of *N*_*m*_ males; based on ${\hat{p}}^{2}$, $2\hat{p}\hat{q}$ and ${\hat{q}}^{2}$, regenerate the genotypes of *N*_*f*_ females;
5. Calculate the value of LRT_0_ based on the new *N*_*m*_ males and *N*_*f*_ females, denoted by ${\text{LRT}}_{0}^{*}$;
6. Repeat Steps 4 and 5 *B* times, which results in *B* test statistics ${\text{LRT}}_{0}^{1*}$, ${\text{LRT}}_{0}^{2*}$, …, ${\text{LRT}}_{0}^{B*}$;
7. The *P*-value of the original LRT_0_ can be estimated as
  $$\hat{P}-\text{value}=\frac{1}{B}\sum_{i=1}^{B}I_{\left\{{LRT}_{0}^{i*}>{\text{LRT}}_{0}\right\}}.$$

For LRT_2*b*_, we can conduct the steps similar to those mentioned above. Firstly, after obtaining the value of LRT_2_, calculate the frequencies of three genotypes *M*_1_ *M*_1_, *M*_1_ *M*_2_ and *M*_2_ *M*_2_ in females under *H*_02_ in the following: ${\hat{p}}_{f}^{2}$, $2{\hat{p}}_{f}{\hat{q}}_{f}$ and ${\hat{q}}_{f}^{2}$, respectively, with ${\hat{p}}_{f}=\left(2n_{2f}+n_{1f}\right)/\left(2N_{f}\right)$. The alleles of *N*_*m*_ males stay the same as the original sample and only regenerate the genotypes of *N*_*f*_ females according to ${\hat{p}}_{f}^{2}$, $2{\hat{p}}_{f}{\hat{q}}_{f}$ and ${\hat{q}}_{f}^{2}$. Then, carry out the similar procedures of Steps 4–7 and we can obtain the the estimated *P*-value of LRT_2_.

### Software implementation

We have written the XHWE software with R (http://www.r-project.org), which includes the eight test statistics: LRT_0_, LRT_0*b*_, LRT_1_, LRT_2_, LRT_2*b*_, *Z*_0_, *Z*_1_ and *Z*_2_. The R package named XHWE is available on CRAN (http://cran.r-project.org/web/packages/XHWE/). The initial values of *p*_*m*_, *p*_*f*_, *p* and *ρ* in the EM algorithms are taken to be *n*_1*m*_/*N*_*m*_, (2*n*_2*f*_ + *n*_1*f*_)/(2*N*_*f*_), (*n*_1*m*_ + 2*n*_2*f*_ + *n*_1*f*_)/(*N*_*m*_ + 2*N*_*f*_) and 0.02, respectively. The convergence criterion is that the absolute differences between the estimates of the parameters at two consecutive iterations are all less than 10^−7^ for the LRT-type statistics. The default maximum number of iterations is 1000. The input data file is the standard pedigree data. The XHWE software only uses the founders with genotypes available in it and will analyze marker loci one by one. The software outputs the values of all the test statistics and the corresponding *P*-values. Also, the XHWE software outputs the estimates of all the parameters under both the null and alternative hypotheses for each test statistic. The parameter estimates under the alternative hypothesis for the LRT-type test statistics are the same. However, under the respective null hypotheses of the LRT-type test statistics, the estimates may be different. It should be noted that the estimates of *p*_*m*_ and *p*_*f*_ under the null hypothesis of *H*_02_ in this article and those in zheng et al. [14] are the same, respectively. The output results will be automatically saved in the text file named “results.txt”.

### Simulation settings

Simulation study is conducted to assess the performance of the proposed LRT_0_, LRT_0*b*_, LRT_1_, LRT_2_, LRT_2*b*_ and *Z*_0_ test statistics and to compare them with the existing *Z*_1_ and *Z*_2_ under various simulation settings which are similar to those in zheng et al. [14]. The allele frequency *p*_*m*_ in males takes two values: 0.3 and 0.5. When *p*_*m*_ is fixed, the value of *p*_*f*_ in females is taken as *p*_*f*_ = *p*_*m*_ + *ϵ*, where *ϵ* = 0, ±0.04 and ±0.05. The inbreeding coefficient *ρ* in females is set at 0 to 0.1 in increment of 0.05. The sample size is taken as 800 and 1200 with the ratio *r* = *N*_*m*_ : *N*_*f*_ being 2:1, 1.5:1, 1:1, 1:1.5 and 1:2. As mentioned earlier, when *p*_*m*_ = *p*_*f*_ and *ρ* = 0, the size of all the eight test statistics is simulated; when *p*_*m*_ = *p*_*f*_ and *ρ* > 0, the size of LRT_1_ and *Z*_1_ is gotten; when *p*_*m*_ ≠ *p*_*f*_ and *ρ* = 0, the size of LRT_2_, LRT_2*b*_ and *Z*_2_ is obtained. Otherwise, we simulate the corresponding powers. In addition, it should be noted that for the fixed sample size (800 or 1200) simulated above, the powers of all the three test statistics LRT_2_, LRT_2*b*_ and *Z*_2_ for *H*_02_ : *ρ* = 0 are not so large, from our simulation results below. On the other hand, these three test statistics only use female individuals. As such, we further obtain the sample size *N*_*f*_ required for LRT_2*b*_ to gain 80% simulated power and then simulate the size and powers of LRT_2_, LRT_2*b*_ and *Z*_2_ under this sample size. To investigate how population structure affects the proposed methods, we also consider the following population stratification model with two subpopulations in our simulation study. *p*_*m*_ = 0.3 (0.5), *p*_*f*_ = *p*_*m*_ + *ϵ*, *ϵ* = 0, ±0.04 and ±0.05 in the first (second) subpopulation and the *ϵ* values are respectively denoted by *ϵ*_1_ and *ϵ*_2_. Assume that *ρ* = 0 in each subpopulation, and the ratio of each subpopulation constructing the population is set to 0.5. The sample size is taken to be 1800, where each individual is a female or a male with equal probability. Note that under population stratification, the null hypothesis *H*_0_: *p*_*m*_ = *p*_*f*_ and *ρ* = 0 is generally not true. Thus, we use the population stratification model to study the powers of the proposed methods. The significance level is fixed at 5% and 10000 replications are simulated under each simulation setting. For LRT_0*b*_ and LRT_2*b*_ via parametric bootstrap, *B* is set to be 1000. Finally, to compare the efficiency of the parameter estimates of the proposed EM algorithms with those in zheng et al. [14] for each simulation setting, we use the RMSEs and biases to assess the accuracy of the parameter estimates, where $\text{RMSE}=\sqrt{{\left[\text{Bias}\left(\hat{\beta }\right)\right]}^{2}+Var\left(\hat{\beta }\right)}$ and $\text{Bias}=E(\hat{\beta })-\beta$, and *β* is the parameter which needs to estimate.

## Results

### Simulation results

Table 1 lists the simulated size of LRT_0_, LRT_0*b*_, LRT_1_, LRT_2_, LRT_2*b*_, *Z*_0_, *Z*_1_ and *Z*_2_ under *H*_0_ : *p*_*m*_ = *p*_*f*_ = *p* and *ρ* = 0 with *N* = 800 and 1200 and *p* = 0.3 and 0.5 for different values of *r* = *N*_*m*_ : *N*_*f*_. According to the table, the size of LRT_1_, *Z*_0_, *Z*_1_ and *Z*_2_ is close to the nominal 5% level, while the size of LRT_0_ and LRT_2_ is too conservative. However, after the parametric bootstrap technique, LRT_0*b*_ and LRT_2*b*_ stay close to the nominal 5% level.

**Table 1:** Simulated size (in %) of LRT_0_, LRT_0*b*_, LRT_1_, LRT_2_, LRT_2*b*_, *Z*_0_, *Z*_1_ and *Z*_2_ under *H*_0_ : *p*_*m*_ = *p*_*f*_ = *p* and *ρ* = 0 with *N* = 800 and 1200 for different values of *r* and *p*.

*N*	*r*	*p*	LRT_0_	LRT_0*b*_	LRT_1_	LRT_2_	LRT_2*b*_	*Z*_0_	*Z*_1_	*Z*_2_
800	2:1	0.3	3.01	4.81	5.02	1.91	4.87	4.83	5.22	4.83
	2:1	0.5	2.98	4.97	5.02	2.28	4.99	5.13	5.19	5.13
	1.5:1	0.3	2.92	4.81	4.74	2.19	4.75	4.99	4.93	4.99
	1.5:1	0.5	3.07	4.93	4.83	2.22	4.98	5.02	4.99	5.02
	1:1	0.3	3.17	5.05	4.74	2.59	5.36	4.82	4.81	4.82
	1:1	0.5	3.30	4.99	5.33	2.40	5.20	5.16	5.34	5.16
	1:1.5	0.3	3.05	5.18	5.09	2.40	5.11	5.09	5.28	5.09
	1:1.5	0.5	3.39	5.18	5.03	2.49	5.34	5.19	5.07	5.19
	1:2	0.3	3.13	4.89	4.77	2.18	4.81	5.13	4.89	5.13
	1:2	0.5	3.12	4.85	4.65	2.32	5.13	5.23	4.78	5.23
1200	2:1	0.3	3.42	5.31	4.84	2.35	5.07	5.47	4.97	5.47
	2:1	0.5	3.45	5.38	4.76	2.48	5.15	5.38	5.01	5.38
	1.5:1	0.3	2.91	4.84	4.84	2.30	5.12	4.83	5.02	5.16
	1.5:1	0.5	3.36	5.31	5.29	2.45	5.35	5.38	5.42	5.38
	1:1	0.3	2.88	4.77	5.05	2.15	4.73	4.73	5.18	4.73
	1:1	0.5	3.04	5.07	5.22	2.01	4.79	4.94	5.30	4.94
	1:1.5	0.3	2.93	4.97	4.75	2.25	4.98	4.98	4.87	4.98
	1:1.5	0.5	3.08	4.83	5.06	2.24	4.86	4.87	5.12	4.87
	1:2	0.3	3.13	4.98	4.83	2.39	5.31	5.03	4.92	5.03
	1:2	0.5	3.24	5.06	4.86	2.54	5.38	5.05	4.91	5.05

**Fig 1:**
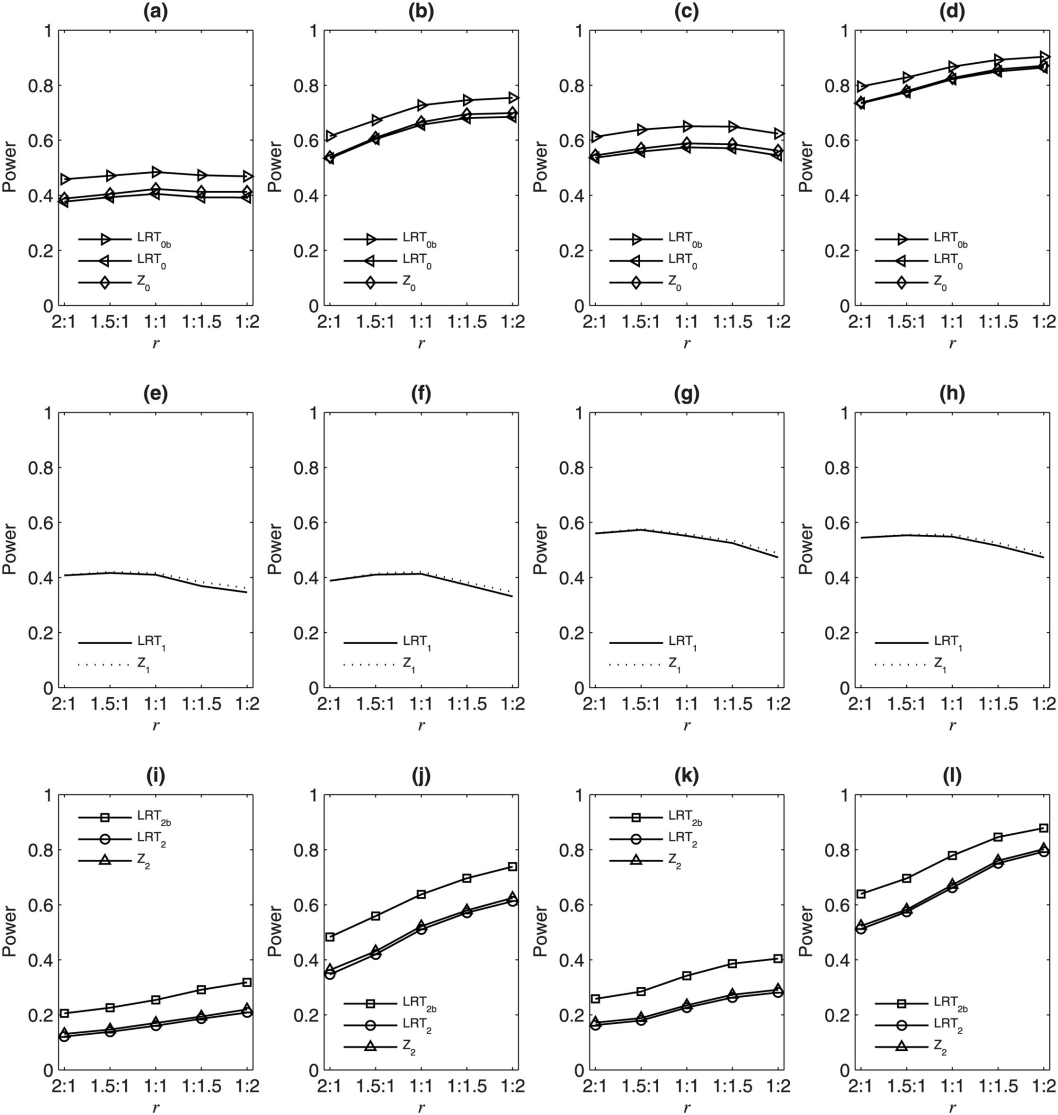
Simulated powers of LRT_0_, LRT_0*b*_, LRT_1_, LRT_2_, LRT_2*b*_, *Z*_0_, *Z*_1_ and *Z*_2_ against *r* = *N*_*m*_ : *N*_*f*_ under *H*_1_ : *p*_*m*_ ≠ *p*_*f*_ and *ρ* > 0 based on 10000 replicates with *p*_*m*_ = 0.3 and *p*_*f*_ = 0.35. In the first column: *ρ* = 0.05 and *N* = 800; in the second column: *ρ* = 0.1 and *N* = 800; in the third column: *ρ* = 0.05 and *N* = 1200; in the fourth column: *ρ* = 0.1 and *N* = 1200. In the first row, the powers of LRT_0_, LRT_0*b*_ and *Z*_0_ for *H*_0_ : *p*_*m*_ = *p*_*f*_ and *ρ* = 0; in the second row, the powers of LRT_1_ and *Z*_1_ for *H*_01_ : *p*_*m*_ = *p*_*f*_; in the third row, the powers of LRT_2_, LRT_2*b*_ and *Z*_2_ for *H*_02_ : *ρ* = 0.

Fig 1 gives the simulated powers of the eight test statistics against *r* under *H*_1_ : *p*_*m*_ ≠ *p*_*f*_ and *ρ* > 0 for different values of *ρ* (0.05 and 0.1) and *N* (800 and 1200), having *p*_*m*_ = 0.3 and *p*_*f*_ = 0.35. It is shown in the figure that LRT_0*b*_ is more powerful than LRT_0_ and *Z*_0_, and LRT_0_ and *Z*_0_ have the similar performance in power (Fig 1a-1d in the first row), regarded of the inbreeding coefficient *ρ*, the sample size *N* and the ratio *r*. LRT_1_ and *Z*_1_ have almost the same performance in power (Fig 1e-1h in the second row). LRT_2*b*_ has much more power than LRT_2_ and *Z*_2_, and LRT_2_ is a little less powerful than *Z*_2_ (Fig 1i-1l in the third row). The powers of LRT_1_ and *Z*_1_ are not so affected by the different values of *r*, while LRT_0_, LRT_0*b*_, *Z*_0_, LRT_2*b*_, LRT_2_ and *Z*_2_ are more and more powerful with the number of female individuals increasing (*r* changing from 2:1 to 1:2) when other parameters are fixed. We also find that the powers of LRT_0_, LRT_0*b*_, *Z*_0_, LRT_2_, LRT_2*b*_ and *Z*_2_ appear great reaction to the different values of *ρ* when *N* is fixed. Specially, their powers under *ρ* = 0.1 (subplots in the second and fourth columns, respectively) are much larger than those under *ρ* = 0.05 (subplots in the first and third columns, respectively). However, the powers of LRT_1_ and *Z*_1_ are almost not influenced by *ρ*. Further, it can be seen in Fig 1 that LRT_0_, LRT_0*b*_ and *Z*_0_ with two degrees of freedom (subplots in the first row) are much more powerful than LRT_1_, *Z*_1_, LRT_2_, LRT_2*b*_ and *Z*_2_ with one degree of freedom (subplots in the second and third rows). This is because the true model is *p*_*m*_ ≠ *p*_*f*_ and *ρ* > 0. In addition, when the sample size changes from 800 (subplots in the first and second columns) to 1200 (subplots in the third and fourth columns), all the test statistics are much more powerful.

**Fig 2:**
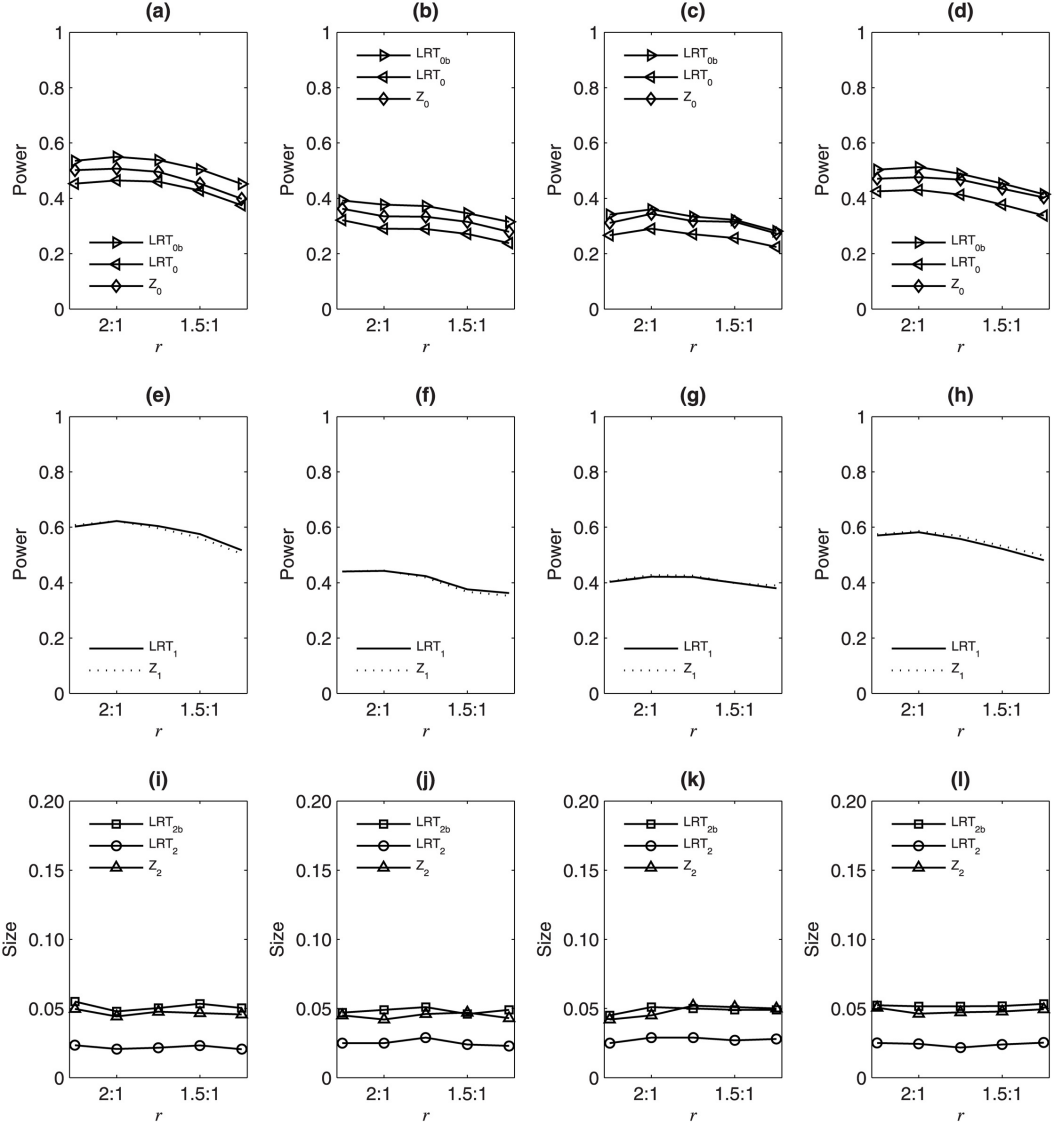
Simulated size/powers of LRT_0_, LRT_0*b*_, LRT_1_, LRT_2_, LRT_2*b*_, *Z*_0_, *Z*_1_ and *Z*_2_ against *r* = *N*_*m*_ : *N*_*f*_ under *H*_02_ : *ρ* = 0 based on 10000 replicates with *p*_*m*_ = 0.3 and *N* = 1200. In the first column: *p*_*f*_ = 0.25; in the second column: *p*_*f*_ = 0.26; in the third column: *p*_*f*_ = 0.34; in the fourth column: *p*_*f*_ = 0.35. In the first row, the powers of LRT_0_, LRT_0*b*_ and *Z*_0_ for *H*_0_ : *p*_*m*_ = *p*_*f*_ and *ρ* = 0; in the second row, the powers of LRT_1_ and *Z*_1_ for *H*_01_ : *p*_*m*_ = *p*_*f*_; in the third row, the size of LRT_2_, LRT_2*b*_ and *Z*_2_ for *H*_02_ : *ρ* = 0.

Fig 2 displays the simulated size/powers of the eight test statistics against *r* under *H*_02_ : *ρ* = 0 for different values of *p*_*f*_, having *p*_*m*_ = 0.3 and *N* = 1200. The results in the third row of the figure are the size of LRT_2_, LRT_2*b*_ and *Z*_2_, while those in the first and the second rows of the figure are the powers of LRT_0_, LRT_0*b*_ and *Z*_0_, and those of LRT_1_ and *Z*_1_, respectively. It is shown in the figure that the size of LRT_2*b*_ and *Z*_2_ maintains close to the nominal 5% level, while LRT_2_ is too conservative. As for the tests for *H*_01_ : *p*_*m*_ = *p*_*f*_, LRT_1_ and *Z*_1_ almost have the same simulated power just like Fig 1. On the other hand, the powers of LRT_0_, LRT_0*b*_, *Z*_0_, LRT_1_ and *Z*_1_ are not so affected by the ratio *r*. However, their powers are greatly influenced by the absolute difference |*ϵ*| = |*p*_*m*_ − *p*_*f*_|. Specifically, their powers under *p*_*f*_ = 0.25 and *p*_*f*_ = 0.35 are much larger than those under *p*_*f*_ = 0.26 and *p*_*f*_ = 0.34. In addition, when the simulation setting is fixed, LRT_1_ and *Z*_1_ with one degree of freedom are a little more powerful than LRT_0_, LRT_0*b*_ and *Z*_0_ with two degrees of freedom, because the true model is *p*_*m*_ ≠ *p*_*f*_ and *ρ* = 0. By comparing Fig 2d (*ρ* = 0), Fig 1c (*ρ* = 0.05) and Fig 1d (*ρ* = 0.1) under *N* = 1200, *p*_*m*_ = 0.3 and *p*_*f*_ = 0.35, LRT_0_, LRT_0*b*_ and *Z*_0_ are more and more powerful with *ρ* increasing.

Figs A–G in S1 File show the corresponding results under other simulation settings with *p*_*m*_ ≠ *p*_*f*_ and *ρ* > 0, which are similar to those in Fig 1. Figs H and I in S1 File plot the corresponding results under *p*_*m*_ = *p*_*f*_ and *ρ* > 0, and Figs J–L in S1 File give the corresponding results under *p*_*m*_ ≠ *p*_*f*_ and *ρ* = 0. The more details refer to S1 File.

Table 2 shows the simulated size of LRT_2_, LRT_2*b*_ and *Z*_2_ for *H*_02_ : *ρ* = 0 for different values of *p*_*f*_, having *N*_*m*_ = 0 under the sample sizes *N*_*f*_ required for LRT_2*b*_ to obtain 80% simulated power. Table 3 lists the simulated powers under these sample sizes for different values of *p*_*f*_, having *ρ* = 0.05 and 0.1. From Table 2, we can see that the type I error rates of LRT_2_, LRT_2*b*_ and *Z*_2_ are close to the nominal significance level of 5%. It is shown in Table 3 that the power of LRT_2*b*_ attains to about 80%, and the difference in power between LRT_2*b*_ and *Z*_2_ is about 10%.

**Table 2:** Simulated size (in %) of LRT_2_, LRT_2*b*_ and *Z*_2_, having *N*_*m*_ = 0 and *ρ* = 0.

*N*_*f*_	*p*_*f*_	**LRT_2_**	**LRT_2*b*_**	*Z*_2_
2500	0.20	2.27	5.18	4.84
	0.25	2.27	4.96	4.89
	0.30	2.43	5.15	5.13
	0.35	2.31	4.99	5.14
	0.40	2.25	4.96	4.70
	0.45	2.43	5.05	4.93
	0.50	2.54	5.03	5.06
	0.55	2.38	5.26	4.86
	0.60	2.49	5.11	5.10
650	0.20	2.14	4.98	4.67
	0.25	2.03	4.62	4.82
	0.30	2.53	4.91	5.20
	0.35	2.45	5.05	5.11
	0.40	2.18	4.72	4.54
	0.45	2.04	4.84	4.65
	0.50	2.48	4.98	5.14
	0.55	2.30	4.74	5.12
	0.60	2.27	5.01	4.77

**Table 3:** Simulated powers (in %) of LRT_2_, LRT_2*b*_ and *Z*_2_, having *N*_*m*_ = 0.

*N*_*f*_	*ρ*	*p*_*f*_	**LRT_2_**	**LRT_2*b*_**	*Z*_2_
2500	0.05	0.20	68.2	79.0	69.7
	0.05	0.25	69.1	79.4	69.9
	0.05	0.30	69.4	79.9	70.2
	0.05	0.35	69.8	80.2	70.3
	0.05	0.40	70.0	80.6	70.4
	0.05	0.45	69.3	79.9	69.6
	0.05	0.50	70.6	80.8	71.5
	0.05	0.55	71.2	81.0	71.5
	0.05	0.60	70.2	80.6	70.5
650	0.10	0.20	68.1	78.9	70.3
	0.10	0.25	69.2	79.9	70.8
	0.10	0.30	69.9	80.9	71.0
	0.10	0.35	70.3	80.5	71.1
	0.10	0.40	71.1	81.1	71.8
	0.10	0.45	71.9	81.9	72.5
	0.10	0.50	71.1	81.6	72.1
	0.10	0.55	70.7	81.4	71.3
	0.10	0.60	70.8	81.9	71.6

Tables A–J in S1 File list the RMSEs and biases of the estimates of *p*_*m*_, *p*_*f*_, the pooled allele frequency *p* and *ρ* for different values of *p*_*m*_, *p*_*f*_, *ρ*, *r* and *N*. It should be noted that the estimate of *p*_*m*_ based on the EM algorithm is the same as zheng et al. [14]. Further, the estimates ${\hat{p}}_{f1}$ and ${\hat{p}}_{01}$ of *p*_*f*_ and *p* based on the EM algorithms have the similar RMSEs and biases as those from zheng et al. [14], respectively. However, when we focus on the estimate of *ρ*, we find that although the biases of ${\hat{\rho }}_{1}$ and ${\hat{\rho }}_{01}$ based on the EM algorithms are larger than ${\hat{\rho }}_{z}$ in zheng et al. [14] for some cases, the RMSEs of ${\hat{\rho }}_{1}$ and ${\hat{\rho }}_{01}$ are smaller than ${\hat{\rho }}_{z}$ for all the simulation settings.

Table 4 displays the simulated size/powers of LRT_0_, LRT_0*b*_, LRT_1_, LRT_2_, LRT_2*b*_, *Z*_0_, *Z*_1_ and *Z*_2_ under the population stratification model. When *ϵ*_1_ = *ϵ*_2_ = 0, the size of LRT_1_ and *Z*_1_ is obtained. Further, note that the ratios of two subpopulations in the whole population are equal. As such, *ϵ*_1_ = −*ϵ*_2_ will also cause the size of LRT_1_ and *Z*_1_. Under other simulation settings, we get the powers of the eight test statistics. To investigate whether or not the population stratification model causes excess homozygosity, we save the values of 10000 *ρ* estimates for each estimation method (${\hat{\rho }}_{1}$, ${\hat{\rho }}_{01}$ or ${\hat{\rho }}_{z}$). Then, calculate the corresponding mean and standard deviation (SD), which are also listed in Table 4. The results show that the population stratification model indeed leads to the positive inbreeding coefficient (i.e., excess homozygosity), which is consistent with Overall and Nichols [24]. The mean $\hat{\rho }$ values (${\hat{\rho }}_{1}$ and ${\hat{\rho }}_{01}$) using the EM algorithm are a little larger than ${\hat{\rho }}_{z}$ proposed in zheng et al. [14], while ${\hat{\rho }}_{1}$ and ${\hat{\rho }}_{01}$ have less standard deviation. On the other hand, the size of LRT_1_ and *Z*_1_ is close to the nominal significance level of 5%. The power of LRT_0*b*_ is larger than LRT_0_ and *Z*_0_, and LRT_0_ and *Z*_0_ have the similar powers, irrespective of the *ϵ*_1_ and *ϵ*_2_ values. LRT_1_ and *Z*_1_ have almost the same powers. LRT_2*b*_ is much more powerful than LRT_2_ and *Z*_2_, and the power of LRT_2_ is a little smaller than *Z*_2_. If *ϵ*_1_ is fixed and *ϵ*_2_ is changed, the *ρ* estimate increases with *ϵ*_2_ increasing, and hence LRT_2_, LRT_2*b*_ and *Z*_2_ are more and more powerful; if *ϵ*_2_ is fixed and *ϵ*_1_ is changed, the *ρ* estimate decreases with *ϵ*_1_ increasing, and hence LRT_2_, LRT_2*b*_ and *Z*_2_ are less and less powerful. This may be caused by *p*_*m*_ being taken to be 0.3 and 0.5 in the first and second subpopulations, respectively.

**Table 4:** Mean and standard deviation (SD) of *ρ* estimates over 10000 replications, and simulated size/powers (in %) of LRT_0_, LRT_0*b*_, LRT_1_, LRT_2_, LRT_2*b*_, *Z*_0_, *Z*_1_ and *Z*_2_ under population stratification model.

*ϵ*		${\hat{\rho }}_{1}$		${\hat{\rho }}_{01}$		${\hat{\rho }}_{z}$		Power							
*ϵ*_1_ ^a^	*ϵ*_2_ ^b^	Mean	SD	Mean	SD	Mean	SD	LRT_0_	LRT_0*b*_	LRT_1_	LRT_2_	LRT_2*b*_	*Z*_0_	*Z*_1_	*Z*_2_
-0.05	-0.05	0.043	0.031	0.046	0.032	0.042	0.034	72.3	78.6	72.1	24.1	37.0	72.6	71.5	25.0
	-0.04	0.050	0.031	0.052	0.032	0.049	0.034	67.8	74.4	63.4	31.0	43.9	67.7	63.0	31.6
	0.00	0.067	0.032	0.068	0.032	0.067	0.033	54.8	63.4	22.7	51.7	65.2	55.3	22.5	52.5
	0.04	0.088	0.034	0.088	0.034	0.087	0.034	65.0	72.3	4.7	73.6	82.8	65.6	4.7	74.5
	0.05	0.093	0.035	0.093	0.035	0.093	0.035	70.9	76.3	4.1	78.7	86.2	71.3	4.1	79.0
-0.04	-0.05	0.039	0.029	0.041	0.030	0.037	0.033	60.5	69.0	63.2	19.2	28.5	60.9	63.2	19.8
	-0.04	0.045	0.030	0.046	0.031	0.043	0.033	57.6	63.8	50.5	25.6	37.6	57.6	49.9	26.2
	0.00	0.061	0.033	0.062	0.033	0.060	0.034	42.2	51.1	17.0	44.5	57.7	42.6	17.0	45.0
	0.04	0.081	0.033	0.081	0.033	0.081	0.034	57.4	64.0	4.2	65.9	77.9	58.1	4.3	66.4
	0.05	0.088	0.033	0.088	0.033	0.088	0.033	65.2	72.5	5.0	74.7	85.2	65.8	5.0	75.7
0.00	-0.05	0.029	0.027	0.030	0.027	0.025	0.033	21.8	29.0	23.0	11.0	19.0	22.5	22.9	12.1
	-0.04	0.030	0.027	0.031	0.027	0.026	0.033	18.9	24.6	17.3	12.5	20.0	19.6	17.2	12.9
	0.00	0.043	0.029	0.043	0.029	0.041	0.032	17.7	24.0	4.3	23.1	34.9	18.2	4.3	23.9
	0.04	0.058	0.032	0.058	0.032	0.058	0.033	42.3	50.6	19.0	40.0	54.1	43.1	19.0	40.6
	0.05	0.063	0.032	0.063	0.032	0.063	0.033	49.4	57.4	23.3	46.2	59.5	50.1	23.5	46.9
0.04	-0.05	0.019	0.023	0.020	0.023	0.012	0.032	6.7	9.4	6.8	4.8	10.0	7.6	6.9	6.1
	-0.04	0.022	0.024	0.022	0.025	0.014	0.034	6.3	9.0	4.7	7.1	11.7	7.0	4.7	7.7
	0.00	0.031	0.028	0.031	0.028	0.026	0.034	18.0	23.7	17.5	11.8	20.7	18.9	17.5	12.8
	0.04	0.041	0.031	0.041	0.031	0.039	0.034	52.1	61.9	51.8	21.2	32.0	52.7	52.1	21.5
	0.05	0.046	0.031	0.047	0.031	0.045	0.034	61.7	69.5	58.6	27.1	38.9	62.1	58.8	27.6
0.05	-0.05	0.018	0.023	0.018	0.023	0.008	0.034	4.1	7.2	4.9	4.5	8.4	5.2	5.0	5.5
	-0.04	0.020	0.023	0.020	0.023	0.011	0.034	6.0	8.4	5.2	4.2	10.1	7.5	5.2	5.9
	0.00	0.028	0.028	0.028	0.028	0.022	0.035	22.1	28.2	24.1	10.4	16.9	22.9	24.2	11.2
	0.04	0.036	0.028	0.037	0.028	0.034	0.032	59.5	65.7	61.6	17.0	27.1	60.0	61.9	17.7
	0.05	0.042	0.031	0.043	0.030	0.040	0.033	68.1	74.1	67.2	22.6	32.1	68.3	67.6	23.4

^a^ *ϵ* in the first subpopulation.

^b^ *ϵ* in the second subpopulation.

### Application to RA data

We apply the proposed methods to the RA dataset from North American Rheumatoid Arthritis Consortium for studying their practicability, which is available from Genetic Analysis Workshop 15. In this dataset, there are 1217 families. Note that many individuals’ genotypes are missing. On the other hand, to obtain a sample of which all the individuals are independent, we only select the available founders in this dataset, which results in a sample composed of 369 founders (*N*_*m*_ = 112 and *N*_*f*_ = 257) in the analysis. 293 SNP markers on X chromosome for each founder are included in this application. The significance level is fixed at *α* = 5%. Table 5 gives the corresponding results based on the *P*-values of LRT_0*b*_, LRT_1_, LRT_2*b*_, *Z*_0_, *Z*_1_ and *Z*_2_. From Table 5, LRT_0*b*_ identified 6 loci which *Z*_0_ did not identify, and *Z*_0_ identified 4 additional loci. One locus is detected by LRT_1_ that is not found by *Z*_1_, and 4 additional loci are detected by *Z*_1_. There are 12 loci identified by LRT_2*b*_, which can not be identified by *Z*_2_, and only 2 additional loci are identified by *Z*_2_. However, there exist multiple testing problems because we simultaneously analyze 293 loci. So, Bonferroni correction is used (*α*′ = 0.05/293 = 1.71 × 10^−4^) and there is no statistically significant result to occur. The more details can be found in Tables K–M in S1 File.

**Table 5:** LRT_0*b*_, LRT_1_, LRT_2*b*_, *Z*_0_, *Z*_1_ and *Z*_2_ results of application to rheumatoid arthritis data at 5% level.

**A. Contingency table showing LRT_0*b*_ and *Z*_0_ results at 5% level**.			
	***P*_*Z*_0__ < 0.05**	***P*_*Z*_0__ ≥ 0.05**	**Total**
***P*_LRT_0*b*__ < 0.05**	11	6	17
***P*_LRT_0*b*__ ≥ 0.05**	4	272	276
**Total**	15	278	293
**B. Contingency table showing LRT_1_ and *Z*_1_ results at 5% level**.			
	***P*_*Z*_1__ < 0.05**	***P*_*Z*_1__ ≥ 0.05**	**Total**
***P*_LRT_1__ < 0.05**	9	1	10
***P*_LRT_1__ ≥ 0.05**	4	279	283
**Total**	13	280	293
**C. Contingency table showing LRT_2*b*_ and *Z*_2_ results at 5% level**.			
	***P*_*Z*_2__ < 0.05**	***P*_*Z*_2__ ≥ 0.05**	**Total**
***P*_LRT_2*b*__ < 0.05**	14	12	26
***P*_LRT_2*b*__ ≥ 0.05**	2	265	267
**Total**	16	277	293

To investigate the computational efficiency of the XHWE software, we implement the code with the default arguments for this dataset (1217 families and 293 SNPs), on a HP 2311f personal computer (Microsoft Windows 7 Enterprise (Service Pack 1), 4GB of RAM and 3.40 GHz Intel(R) Core(TM) i7 Duo processor) and record its computational time. This process needs 977 seconds. Therefore, on the average, the running time for a single SNP is about 3.3 seconds. For the genome-wide case, for example, one would analyze 200000 SNP markers on X chromosome for the family sample of the type mentioned above, which would lead to 1600000 tests for the hypotheses with running time being about 7.6 days on the personal computer of this type.

## Discussion

The existing *Z*_1_ and *Z*_2_ tests were respectively proposed to test for *H*_01_ : *p*_*m*_ = *p*_*f*_ and *H*_02_ : *ρ* = 0. However, we find that there is no simulation study conducted to assess the validity of *Z*_1_ and *Z*_2_ and their performance [14]. Further, there is no existing method to simultaneously test for *H*_0_ : *p*_*m*_ = *p*_*f*_ and *ρ* = 0. Therefore, in this article, we first combine these two test statistics and suggest *Z*_0_ = *Z*_1_ + *Z*_2_ to test for the equality of the frequencies of the same allele in males and females and the zero inbreeding coefficient on X chromosome based on the collected sample, because *Z*_1_ and *Z*_2_ are independent of each other. What’s more, for the purpose of improving the test power, we propose several LRT-type test statistics. Firstly, we write out the likelihood functions under *H*_0_ : *p*_*m*_ = *p*_*f*_ and *ρ* = 0 and *H*_1_ : *p*_*m*_ ≠ *p*_*f*_ or *ρ* > 0 at a single SNP locus on X chromosome, respectively. Then, we obtain the MLEs of the male allele frequency, the female allele frequency and the inbreeding coefficient by the EM algorithms, where we use the RMSE and bias to assess the accuracy of the MLEs of these unknown parameters and construct the corresponding likelihood ratio test (LRT_0_) statistic under the null hypothesis *H*_0_. If *H*_0_ is statistically rejected, we further develop two LRT-type test statistics LRT_1_ and LRT_2_ respectively for *H*_01_ : *p*_*m*_ = *p*_*f*_ and *H*_02_ : *ρ* = 0. Note that LRT_0_ and LRT_2_ are too conservative from the simulated results. So, we use parametric bootstrap techniques and propose the LRT_0*b*_ and LRT_2*b*_ test statistics. We simulate the data under different parameter settings. Simulation results show that the proposed bootstrap-based methods LRT_0*b*_ and LRT_2*b*_, LRT_1_, *Z*_0_ and the existing *Z*_1_ and *Z*_2_ control the type I error rates well under the respective null hypothesis. Power comparison demonstrates that LRT_0*b*_ is more powerful than both LRT_0_ and *Z*_0_. Under *ρ* > 0, LRT_2*b*_ has much more power than LRT_2_ and *Z*_2_, and LRT_2_ is a little less powerful than *Z*_2_. In addition, LRT_1_ and *Z*_1_ almost have the same power under *p*_*m*_ ≠ *p*_*f*_.

As for the parameter estimates, the estimate of *p*_*m*_ based on the EM algorithm is the same as that in zheng et al. [14]. Further, the estimates ${\hat{p}}_{f1}$ and ${\hat{p}}_{01}$ of *p*_*f*_ and the pooled allele frequency *p* based on the EM algorithms have the RMSE and bias similar to those from zheng et al. [14], respectively. However, although the biases of ${\hat{\rho }}_{1}$ and ${\hat{\rho }}_{01}$ based on the EM algorithms are larger than ${\hat{\rho }}_{z}$ from zheng et al. [14] for some cases, the RMSEs of ${\hat{\rho }}_{1}$ and ${\hat{\rho }}_{01}$ are smaller than ${\hat{\rho }}_{z}$ for all the simulation settings. In addition, the population stratification model indeed causes excess homozygosity, which is consistent with Overall and Nichols [24]. The mean $\hat{\rho }$ values (${\hat{\rho }}_{1}$ and ${\hat{\rho }}_{01}$) using the EM algorithm are a little larger than ${\hat{\rho }}_{z}$ proposed in zheng et al. [14], while ${\hat{\rho }}_{1}$ and ${\hat{\rho }}_{01}$ have less standard deviation.

Note that *ρ* = 0 and *ρ* > 0 in the null and alternative hypotheses of the likelihood ratio test LRT_0_ or LRT_2_, respectively, which causes the “boundary” problem and that the corresponding likelihood ratio test is not expected to follow a *χ* ^2^ distribution [31, 33]. This may be the reason why the size of LRT_0_ and LRT_2_ is too conservative. Therefore, we use parametric bootstrap techniques to obtain the exact distributions of LRT_0_ and LRT_2_ in this article.

Due to the presence of the X chromosome inactivation (XCI) and dosage compensation (DC), association analysis and excess homozygosity tests on X chromosome are more complicated than those on autosomes [34]. In the presence of XCI, only one allele from a pair of alleles in females is expressed [35]. Consequently, if considering a locus with two alleles *M*_1_ and *M*_2_, the effect of the *M*_1_ allele in males should be equivalent to the difference between *M*_2_ *M*_2_ and *M*_1_ *M*_1_ homozygous females. As such, when we conduct analyses based on allele-counting, we must either count each allele twice in males or equivalently count each allele in females as 0.5, reflecting a “dosage compensation” for X inactivation [34]. It should be noted that LRT_2_, LRT_2*b*_ and *Z*_2_ for *H*_02_ : *ρ* = 0 are not affected by XCI and DC because they only use female individuals in the collected sample. Similarly, *Z*_1_ for *H*_01_ : *p*_*m*_ = *p*_*f*_ is also not influenced by XCI and DC because it estimates the allele frequencies and the corresponding variances in males and females, respectively. Thus, *Z*_0_ = *Z*_1_ + *Z*_2_ is still valid when XCI and DC exist. To investigate the effect of XCI and DC on LRT_0_, LRT_0*b*_, LRT_1_ and LRT_1*b*_, where LRT_1*b*_ is the bootstrap version of LRT_1_, we carry out simulation study under several simulation settings in the presence of XCI and DC. The simulation settings and simulation results are listed in Table 6. It is shown in the table that the size of LRT_2*b*_, *Z*_0_, *Z*_1_ and *Z*_2_ stays close to the nominal 5% level and the size of LRT_2_ is still conservative. However, LRT_0_ and LRT_1_ without bootstrap cannot control the size well. Fortunately, the type I error rates of LRT_0*b*_ and LRT_1*b*_ with bootstrap are very close to 5%. Furthermore, LRT_0*b*_ is more powerful than *Z*_0_ almost for all the cases and LRT_1*b*_ and *Z*_1_ almost have the same performance in power. Therefore, in the presence of XCI and DC, LRT_0*b*_, *Z*_1_ and LRT_2*b*_ are recommended. Finally, LRT_0*b*_ and LRT_2*b*_ can deal with samples of small size. However, LRT_0*b*_ and LRT_2*b*_ are based on the parametric bootstrap techniques, which are more computationally intensive.

**Table 6:** Simulated size/powers (in %) of LRT_0_, LRT_0*b*_, LRT_1_, LRT_1*b*_, LRT_2_, LRT_2*b*_, *Z*_0_, *Z*_1_ and *Z*_2_ based on 10000 Monte Carlo replications and 1000 bootstrap replications under X chromosome inactivation and dose compensation, having *p*_*m*_ = 0.3 and the ratio *N*_*m*_ : *N*_*f*_ = 1: 1.

*N*	*ρ*	*p*_*f*_	**LRT_0_**	**LRT_0*b*_**	**LRT_1_**	**LRT_1*b*_**	**LRT_2_**	**LRT_2*b*_**	*Z*_0_	*Z*_1_	*Z*_2_
800	0.00	0.30	6.5	5.0	10.6	4.9	2.3	5.2	4.8	4.8	4.9
	0.05	0.30	17.2	13.3	10.8	4.9	16.2	25.9	13.0	5.0	17.0
	0.10	0.30	44.1	38.5	11.4	5.2	49.3	63.0	41.2	5.3	50.5
	0.00	0.34	31.7	27.3	42.2	29.2	2.5	5.4	23.5	29.7	5.2
	0.05	0.34	41.8	36.5	41.2	27.9	15.8	26.3	31.6	28.4	16.4
	0.10	0.34	65.1	59.5	41.0	28.2	50.9	64.0	58.5	28.5	52.0
	0.00	0.35	43.6	38.7	55.5	41.3	2.2	4.9	33.6	41.9	5.0
	0.05	0.35	54.3	48.9	55.3	41.5	15.8	26.0	42.0	41.9	16.7
	0.10	0.35	72.1	67.3	53.7	40.2	49.9	63.1	64.8	40.6	50.9
1200	0.00	0.30	6.7	4.8	10.9	5.1	2.2	4.8	5.1	5.0	5.5
	0.05	0.30	22.4	18.0	10.8	5.1	23.0	34.3	18.4	5.0	24.3
	0.10	0.30	60.9	54.8	11.2	5.5	67.3	78.5	58.6	5.5	68.3
	0.00	0.34	42.7	37.8	55.0	40.7	2.2	4.9	32.5	41.3	4.9
	0.05	0.34	57.3	52.1	54.4	40.8	22.1	33.6	46.6	41.0	22.9
	0.10	0.34	81.0	76.8	53.0	39.3	67.5	79.3	76.0	39.5	68.7
	0.00	0.35	59.5	53.8	70.6	57.4	2.3	4.9	47.5	57.9	5.0
	0.05	0.35	71.2	66.2	70.3	57.5	22.7	34.3	59.7	57.8	23.8
	0.10	0.35	88.1	84.7	68.5	55.4	67.7	78.8	83.6	56.0	68.7

## Supporting Information

S1 File Supporting Information. Tables A–J, root mean squared errors (RMSE) and biases of estimates of *p*_*m*_, *p*_*f*_ and *ρ* based on EM algorithm and zheng et al. [14] under different simulation settings. Tables K–M, LRT_0_, LRT_0*b*_, *Z*_0_, LRT_1_, *Z*_1_, LRT_2_, LRT_2*b*_, and *Z*_2_ results of application to rheumatoid arthritis data, respectively. Figs A–L, simulated size/powers of LRT_0_, LRT_0*b*_, LRT_1_, LRT_2_, LRT_2*b*_, *Z*_0_, *Z*_1_ and *Z*_2_ against *r* = *N*_*m*_ : *N*_*f*_ based on 10000 replicates under different simulation settings.
(PDF)
