# Supplementary material for: Likelihood Ratio Test for Excess Homozygosity at Marker Loci on X Chromosome
Source: PLoS One. 2015 Dec 15;10(12):e0145032. doi: 10.1371/journal.pone.0145032 (PMC4684405; doi:10.1371/journal.pone.0145032)
Supplement: S1 File — Tables A–J, root mean squared errors (RMSE) and biases of estimates of p m, p f and ρ based on EM algorithm and zheng et al. [14] under different simulation settings. Tables K–M, LRT0, LRT0b, Z 0, LRT1, Z 1, LRT2, LRT2b, and Z 2 results of application to rheumatoid arthritis data, respectively. Figs A–L, simulated size/powers of LRT0, LRT0b, LRT1, LRT2, LRT2b, Z 0, Z 1 and Z 2 against r = N m : N f based on 10000 replicates under different simulation settings. (PDF) [file pone.0145032.s001.pdf]

Supporting Information

for

"Likelihood Ratio Test for Excess Homozygosity at Marker Loci on X Chromosome

by

Xiao-Ping You, Qi-Lei Zou, Jian-Long Li, Ji-Yuan Zhou*"

Table A. Root mean squared errors (RMSE) and biases of estimates of p_m, p_f and ρ based on EM algorithm and Zheng et al. [14], having $p_m = 0.3$ and $p_f = 0.25$.

			$\hat{p}_m$		$\hat{p}_{f1}$		$\hat{\rho}_1$		$\hat{p}_f$		$\hat{\rho}_z$	
N	r	ρ	RMSE	Bias ×10000	RMSE	Bias ×10000	RMSE	Bias ×10000	RMSE	Bias ×10000	RMSE	Bias ×10000
800	2:1	0.00	0.020	-1.08	0.019	2.17	0.043	236.99	0.019	2.17	0.062	-21.10
	1.5:1	0.00	0.021	-1.04	0.017	2.16	0.039	214.04	0.017	2.16	0.056	-18.42
	1:1	0.00	0.023	3.34	0.015	0.22	0.035	192.46	0.015	0.22	0.051	-16.70
	1:1.5	0.00	0.026	-1.33	0.014	0.52	0.033	181.82	0.014	0.52	0.046	-6.01
	1:2	0.00	0.028	0.75	0.013	0.22	0.030	163.36	0.013	0.22	0.043	-16.53
	2:1	0.05	0.020	-3.01	0.019	-0.46	0.052	66.36	0.019	-0.46	0.063	-13.39
	1.5:1	0.05	0.021	-0.66	0.018	0.02	0.048	49.63	0.018	0.03	0.057	-11.34
	1:1	0.05	0.023	6.71	0.016	2.36	0.044	32.26	0.016	2.36	0.051	-12.21
	1:1.5	0.05	0.026	-0.14	0.014	1.41	0.042	33.99	0.014	1.41	0.048	-1.11
	1:2	0.05	0.028	0.12	0.014	-2.96	0.040	17.37	0.014	-2.95	0.045	-15.55
	2:1	0.10	0.020	-3.02	0.020	4.04	0.061	-13.05	0.020	4.04	0.065	-31.09
	1.5:1	0.10	0.021	-3.03	0.018	0.65	0.056	-0.13	0.018	0.65	0.059	-11.04
	1:1	0.10	0.023	-0.54	0.016	-0.02	0.052	-3.19	0.016	-0.02	0.053	-8.29
	1:1.5	0.10	0.026	-4.93	0.015	-0.39	0.047	-8.52	0.015	-0.39	0.048	-11.72
	1:2	0.10	0.028	-5.52	0.014	1.31	0.045	-14.47	0.014	1.31	0.045	-16.78
1200	2:1	0.00	0.016	-0.56	0.015	4.08	0.035	197.49	0.015	4.08	0.050	-6.46
	1.5:1	0.00	0.017	-0.82	0.014	-0.41	0.031	174.54	0.014	-0.41	0.045	-12.93
	1:1	0.00	0.019	-2.38	0.013	-0.05	0.029	159.73	0.013	-0.05	0.041	-7.82
	1:1.5	0.00	0.021	2.03	0.011	-0.05	0.026	147.00	0.011	-0.05	0.037	-3.09
	1:2	0.00	0.023	0.54	0.011	-0.30	0.024	134.35	0.011	-0.30	0.035	-11.96
	2:1	0.05	0.016	0.78	0.016	0.05	0.044	24.59	0.016	0.05	0.051	-23.75
	1.5:1	0.05	0.017	-1.06	0.014	-1.13	0.041	19.84	0.014	-1.13	0.047	-14.68
	1:1	0.05	0.019	-0.03	0.013	-0.38	0.038	19.43	0.013	-0.38	0.042	-5.29
	1:1.5	0.05	0.021	0.28	0.012	-0.54	0.036	10.30	0.012	-0.54	0.039	-7.99
	1:2	0.05	0.023	2.01	0.011	-2.22	0.034	5.84	0.011	-2.22	0.036	-7.94
	2:1	0.10	0.016	-1.00	0.016	2.07	0.051	-11.04	0.016	2.07	0.052	-16.48
	1.5:1	0.10	0.017	-2.19	0.015	-3.18	0.047	-12.70	0.015	-3.18	0.048	-15.57
	1:1	0.10	0.019	0.42	0.013	2.26	0.042	-8.08	0.013	2.26	0.042	-9.35
	1:1.5	0.10	0.021	-0.31	0.012	-1.62	0.039	-10.34	0.012	-1.62	0.039	-10.88
	1:2	0.10	0.023	-1.91	0.011	-0.31	0.037	-8.75	0.011	-0.31	0.037	-9.24

Table B. Root mean squared errors (RMSE) and biases of estimates of p_m, p_f and ρ based on EM algorithm and Zheng et al. [14], having $p_m = 0.3$ and $p_f = 0.26$.

			$\hat{p}_m$		$\hat{p}_{f1}$		$\hat{\rho}_1$		$\hat{p}_f$		$\hat{\rho}_z$	
N	r	ρ	RMSE	Bias ×10000	RMSE	Bias ×10000	RMSE	Bias ×10000	RMSE	Bias ×10000	RMSE	Bias ×10000
800	2:1	0.00	0.020	0.62	0.019	-1.26	0.042	230.38	0.019	-1.26	0.061	-27.80
	1.5:1	0.00	0.021	-0.63	0.018	0.75	0.039	215.72	0.018	0.75	0.056	-13.26
	1:1	0.00	0.023	2.63	0.016	-0.48	0.036	200.02	0.016	-0.49	0.051	-3.42
	1:1.5	0.00	0.026	-1.33	0.015	-0.07	0.032	181.27	0.015	-0.07	0.046	-7.99
	1:2	0.00	0.028	2.82	0.014	-1.82	0.031	172.47	0.014	-1.82	0.043	-1.90
	2:1	0.05	0.020	2.47	0.020	-2.08	0.051	56.22	0.020	-2.08	0.062	-20.95
	1.5:1	0.05	0.021	-2.28	0.018	-0.10	0.048	52.89	0.018	-0.10	0.057	-7.12
	1:1	0.05	0.023	0.77	0.016	-2.40	0.044	33.61	0.016	-2.40	0.051	-10.93
	1:1.5	0.05	0.026	3.48	0.015	0.59	0.041	19.73	0.015	0.59	0.047	-16.55
	1:2	0.05	0.028	-4.04	0.014	-0.79	0.040	20.83	0.014	-0.79	0.044	-9.13
	2:1	0.10	0.020	-1.53	0.020	-3.57	0.060	-8.04	0.020	-3.57	0.063	-23.68
	1.5:1	0.10	0.021	-3.03	0.019	1.24	0.056	0.17	0.019	1.24	0.058	-10.33
	1:1	0.10	0.023	-0.54	0.017	-0.71	0.050	-3.34	0.017	-0.71	0.051	-8.04
	1:1.5	0.10	0.026	-1.94	0.015	-1.42	0.046	-5.55	0.015	-1.42	0.047	-8.15
	1:2	0.10	0.028	0.21	0.014	-1.80	0.044	-3.52	0.014	-1.80	0.045	-5.64
1200	2:1	0.00	0.016	0.81	0.016	-1.89	0.035	194.80	0.016	-1.89	0.050	-10.12
	1.5:1	0.00	0.017	1.27	0.014	0.05	0.032	181.06	0.014	0.05	0.046	-4.42
	1:1	0.00	0.019	1.33	0.013	-0.43	0.029	160.23	0.013	-0.43	0.041	-5.25
	1:1.5	0.00	0.021	-0.21	0.012	-1.33	0.026	145.55	0.012	-1.33	0.037	-6.55
	1:2	0.00	0.023	-1.62	0.011	-1.46	0.025	136.56	0.011	-1.46	0.035	-9.76
	2:1	0.05	0.016	0.42	0.017	-0.48	0.044	35.26	0.017	-0.48	0.051	-11.98
	1.5:1	0.05	0.017	-0.13	0.015	-0.32	0.041	20.90	0.015	-0.32	0.047	-15.48
	1:1	0.05	0.019	-1.67	0.013	0.76	0.038	20.32	0.013	0.76	0.042	-3.90
	1:1.5	0.05	0.021	-1.22	0.012	0.71	0.035	9.91	0.012	0.71	0.038	-7.65
	1:2	0.05	0.023	-1.81	0.012	0.51	0.034	9.50	0.012	0.51	0.036	-4.39
	2:1	0.10	0.016	-0.22	0.017	0.14	0.050	-14.33	0.017	0.14	0.052	-19.75
	1.5:1	0.10	0.017	-1.04	0.015	0.64	0.047	-15.60	0.015	0.64	0.048	-18.71
	1:1	0.10	0.019	1.93	0.014	1.38	0.042	-2.28	0.014	1.38	0.042	-3.70
	1:1.5	0.10	0.021	0.20	0.012	-1.61	0.038	-9.50	0.012	-1.61	0.038	-10.08
	1:2	0.10	0.023	0.36	0.012	-0.94	0.036	-10.13	0.012	-0.94	0.036	-10.48

Table C. Root mean squared errors (RMSE) and biases of estimates of p and ρ under $H_{01} : p_m = p_f = p$, based on EM algorithm and Zheng et al. [14], having $p = 0.3$.

			$\hat{p}_{01}$		$\hat{\rho}_{01}$		$\hat{p}$		$\hat{\rho}_z$	
N	r	ρ	RMSE	Bias ×10000	RMSE	Bias ×10000	RMSE	Bias ×10000	RMSE	Bias ×10000
800	2:1	0.00	0.014	-0.67	0.043	240.31	0.014	-0.70	0.061	-16.91
	1.5:1	0.00	0.014	0.80	0.040	222.14	0.014	0.79	0.057	-17.71
	1:1	0.00	0.013	1.67	0.036	200.15	0.013	1.69	0.050	-2.52
	1:1.5	0.00	0.013	-0.39	0.032	177.29	0.013	-0.39	0.045	-10.84
	1:2	0.00	0.013	1.07	0.030	169.48	0.013	1.08	0.043	-6.35
	2:1	0.05	0.014	1.10	0.052	62.98	0.014	1.07	0.063	-24.21
	1.5:1	0.05	0.014	-1.41	0.049	51.48	0.014	-1.43	0.057	-15.54
	1:1	0.05	0.013	2.09	0.044	34.01	0.013	2.18	0.051	-14.56
	1:1.5	0.05	0.013	-0.46	0.041	20.68	0.013	-0.44	0.046	-16.25
	1:2	0.05	0.013	2.03	0.039	15.49	0.013	2.05	0.044	-15.98
	2:1	0.10	0.014	0.15	0.060	12.38	0.014	0.04	0.063	-13.35
	1.5:1	0.10	0.014	0.61	0.055	-1.55	0.014	0.74	0.057	-17.68
	1:1	0.10	0.014	0.09	0.050	-3.53	0.014	-0.09	0.052	-14.07
	1:1.5	0.10	0.013	0.19	0.046	-2.57	0.013	0.14	0.047	-8.41
	1:2	0.10	0.013	1.80	0.043	-4.74	0.013	1.82	0.044	-8.66
1200	2:1	0.00	0.012	-0.44	0.035	196.82	0.012	-0.48	0.050	-14.06
	1.5:1	0.00	0.011	0.22	0.032	176.74	0.011	0.20	0.045	-14.41
	1:1	0.00	0.011	-0.41	0.028	158.70	0.011	-0.39	0.041	-9.84
	1:1.5	0.00	0.011	1.32	0.025	139.79	0.011	1.32	0.037	-18.22
	1:2	0.00	0.010	1.71	0.025	140.52	0.010	1.70	0.036	-5.18
	2:1	0.05	0.011	0.05	0.044	45.93	0.012	0.04	0.051	-4.29
	1.5:1	0.05	0.011	1.82	0.041	34.56	0.011	1.85	0.046	-2.53
	1:1	0.05	0.011	0.64	0.037	14.14	0.011	0.61	0.041	-12.34
	1:1.5	0.05	0.011	0.15	0.035	13.08	0.011	0.13	0.038	-5.35
	1:2	0.05	0.010	0.34	0.034	9.21	0.010	0.36	0.036	-5.23
	2:1	0.10	0.012	-1.27	0.050	-6.21	0.012	-1.17	0.051	-17.75
	1.5:1	0.10	0.011	-1.53	0.047	-14.31	0.011	-1.56	0.047	-22.48
	1:1	0.10	0.011	-0.63	0.041	-8.49	0.011	-0.61	0.041	-13.02
	1:1.5	0.10	0.011	0.08	0.038	-0.97	0.011	0.11	0.038	-3.48
	1:2	0.10	0.011	-0.68	0.036	-8.92	0.011	-0.67	0.036	-10.69

Table D. Root mean squared errors (RMSE) and biases of estimates of p_m, p_f and ρ based on EM algorithm and Zheng et al. [14], having $p_m = 0.3$ and $p_f = 0.34$.

			$\hat{p}_m$		$\hat{p}_{f1}$		$\hat{\rho}_1$		$\hat{p}_f$		$\hat{\rho}_z$	
N	r	ρ	RMSE	Bias ×10000	RMSE	Bias ×10000	RMSE	Bias ×10000	RMSE	Bias ×10000	RMSE	Bias ×10000
800	2:1	0.00	0.020	-1.99	0.020	1.99	0.042	234.35	0.020	1.99	0.062	-24.01
	1.5:1	0.00	0.021	-2.09	0.019	-2.34	0.039	214.81	0.019	-2.34	0.056	-19.18
	1:1	0.00	0.023	0.57	0.017	0.20	0.034	188.76	0.017	0.20	0.050	-20.93
	1:1.5	0.00	0.026	-1.48	0.015	0.49	0.032	179.24	0.015	0.49	0.045	-3.39
	1:2	0.00	0.028	4.05	0.014	-0.26	0.030	169.93	0.014	-0.26	0.043	-6.77
	2:1	0.05	0.020	0.41	0.021	-3.18	0.051	60.83	0.021	-3.18	0.062	-14.81
	1.5:1	0.05	0.021	-2.76	0.019	1.23	0.048	46.16	0.019	1.23	0.057	-16.29
	1:1	0.05	0.023	-1.94	0.017	0.67	0.043	30.22	0.017	0.67	0.051	-15.62
	1:1.5	0.05	0.026	1.90	0.015	-0.04	0.041	20.71	0.015	-0.04	0.047	-15.43
	1:2	0.05	0.028	-3.38	0.015	-0.05	0.039	11.34	0.015	-0.05	0.044	-18.42
	2:1	0.10	0.020	0.41	0.021	0.64	0.059	-4.21	0.021	0.64	0.062	-18.97
	1.5:1	0.10	0.021	-2.30	0.019	-0.15	0.055	2.64	0.019	-0.15	0.057	-5.94
	1:1	0.10	0.023	1.51	0.017	0.85	0.050	-9.18	0.017	0.85	0.051	-13.99
	1:1.5	0.10	0.026	-2.93	0.016	-1.55	0.046	-6.99	0.016	-1.55	0.046	-9.38
	1:2	0.10	0.028	-3.55	0.015	0.13	0.044	-3.09	0.015	0.13	0.044	-4.68
1200	2:1	0.00	0.016	3.43	0.017	-1.15	0.035	194.47	0.017	-1.15	0.050	-11.51
	1.5:1	0.00	0.017	-0.28	0.015	-0.54	0.032	177.91	0.015	-0.54	0.046	-9.04
	1:1	0.00	0.019	1.69	0.013	0.21	0.028	155.37	0.013	0.21	0.041	-12.53
	1:1.5	0.00	0.021	-0.57	0.012	-1.49	0.026	148.76	0.012	-1.49	0.037	-0.10
	1:2	0.00	0.023	-2.40	0.012	0.90	0.025	139.38	0.012	0.90	0.035	-2.42
	2:1	0.05	0.016	-1.40	0.017	-0.21	0.044	27.91	0.017	-0.21	0.051	-17.33
	1.5:1	0.05	0.017	-0.34	0.015	-0.82	0.041	31.05	0.015	-0.82	0.046	-1.76
	1:1	0.05	0.018	-2.88	0.014	-2.19	0.037	10.44	0.014	-2.19	0.041	-11.44
	1:1.5	0.05	0.021	2.16	0.013	-1.77	0.035	10.31	0.013	-1.77	0.038	-5.49
	1:2	0.05	0.023	-0.83	0.012	0.10	0.033	9.25	0.012	0.10	0.036	-5.08
	2:1	0.10	0.016	1.50	0.017	-1.94	0.050	-16.62	0.017	-1.94	0.051	-22.26
	1.5:1	0.10	0.017	-0.80	0.016	1.02	0.046	-4.30	0.016	1.02	0.046	-6.81
	1:1	0.10	0.019	-0.70	0.014	0.23	0.041	-16.15	0.014	0.24	0.041	-16.92
	1:1.5	0.10	0.021	0.79	0.013	0.40	0.038	-3.71	0.013	0.40	0.038	-4.20
	1:2	0.10	0.023	2.61	0.012	1.62	0.036	-9.65	0.012	1.62	0.036	-9.91

Table E. **Root mean squared errors (RMSE) and biases of estimates of p_m, p_f and ρ based on EM algorithm and Zheng et al. [14], having $p_m = 0.3$ and $p_f = 0.35$.**

			$\hat{p}_m$		$\hat{p}_{f1}$		$\hat{\rho}_1$		$\hat{p}_f$		$\hat{\rho}_z$	
N	r	ρ	RMSE	Bias ×10000	RMSE	Bias ×10000	RMSE	Bias ×10000	RMSE	Bias ×10000	RMSE	Bias ×10000
800	2:1	0.00	0.020	0.81	0.021	-2.37	0.042	231.74	0.021	-2.37	0.062	-31.17
	1.5:1	0.00	0.021	2.68	0.019	-0.73	0.039	216.59	0.019	-0.73	0.056	-14.75
	1:1	0.00	0.023	-1.84	0.017	-0.74	0.035	192.78	0.017	-0.74	0.050	-12.61
	1:1.5	0.00	0.026	-4.94	0.015	1.09	0.032	174.66	0.015	1.09	0.046	-17.43
	1:2	0.00	0.028	7.07	0.015	-1.09	0.030	165.49	0.015	-1.09	0.043	-12.73
	2:1	0.05	0.020	-1.89	0.021	-1.10	0.051	50.42	0.021	-1.10	0.062	-26.63
	1.5:1	0.05	0.021	0.20	0.019	-0.29	0.047	47.11	0.019	-0.29	0.056	-12.09
	1:1	0.05	0.023	1.27	0.017	-1.80	0.044	28.43	0.017	-1.80	0.051	-17.20
	1:1.5	0.05	0.026	0.33	0.016	1.72	0.041	18.78	0.016	1.72	0.046	-13.94
	1:2	0.05	0.028	1.46	0.015	1.35	0.039	23.28	0.015	1.35	0.043	-3.67
	2:1	0.10	0.020	0.76	0.022	-4.29	0.059	-24.41	0.022	-4.29	0.062	-40.26
	1.5:1	0.10	0.021	-0.46	0.020	1.03	0.055	-4.32	0.020	1.03	0.057	-14.40
	1:1	0.10	0.023	-3.18	0.018	2.28	0.050	-9.72	0.018	2.28	0.051	-15.01
	1:1.5	0.10	0.026	-1.53	0.016	-0.27	0.046	-10.54	0.016	-0.27	0.047	-13.32
	1:2	0.10	0.028	0.78	0.015	-2.33	0.043	-14.74	0.015	-2.33	0.044	-16.34
1200	2:1	0.00	0.016	-0.46	0.017	1.21	0.035	198.06	0.017	1.21	0.050	-5.12
	1.5:1	0.00	0.017	-0.74	0.015	-1.87	0.032	177.01	0.015	-1.87	0.045	-6.72
	1:1	0.00	0.019	1.43	0.014	-1.36	0.028	159.86	0.014	-1.36	0.041	-4.09
	1:1.5	0.00	0.021	1.23	0.012	2.44	0.026	144.91	0.012	2.44	0.037	-4.99
	1:2	0.00	0.023	1.57	0.012	0.69	0.025	139.97	0.012	0.69	0.036	-3.49
	2:1	0.05	0.016	1.22	0.017	1.44	0.044	26.92	0.017	1.45	0.051	-18.44
	1.5:1	0.05	0.017	0.43	0.016	2.49	0.040	15.66	0.016	2.49	0.046	-19.58
	1:1	0.05	0.019	3.31	0.014	0.29	0.037	17.40	0.014	0.29	0.041	-5.32
	1:1.5	0.05	0.021	-1.65	0.013	0.63	0.034	11.97	0.013	0.63	0.037	-3.66
	1:2	0.05	0.023	-1.97	0.012	-1.68	0.033	4.84	0.012	-1.68	0.036	-8.79
	2:1	0.10	0.016	1.53	0.018	-0.38	0.050	-4.10	0.018	-0.38	0.051	-8.84
	1.5:1	0.10	0.017	2.62	0.016	-3.15	0.046	-12.16	0.016	-3.15	0.046	-14.94
	1:1	0.10	0.019	3.67	0.014	-1.40	0.041	-15.16	0.014	-1.40	0.042	-16.34
	1:1.5	0.10	0.021	-0.14	0.013	0.48	0.038	-9.57	0.013	0.48	0.038	-9.99
	1:2	0.10	0.023	-1.10	0.012	1.23	0.036	-6.80	0.012	1.23	0.036	-7.04

Table F. Root mean squared errors (RMSE) and biases of estimates of p_m, p_f and ρ based on EM algorithm and Zheng et al. [14], having $p_m = 0.5$ and $p_f = 0.45$.

			$\hat{p}_m$		$\hat{p}_{f1}$		$\hat{\rho}_1$		$\hat{p}_f$		$\hat{\rho}_z$	
N	r	ρ	RMSE	Bias ×10000	RMSE	Bias ×10000	RMSE	Bias ×10000	RMSE	Bias ×10000	RMSE	Bias ×10000
800	2:1	0.00	0.022	0.17	0.022	-0.52	0.041	226.59	0.022	-0.52	0.061	-28.51
	1.5:1	0.00	0.023	1.70	0.020	2.43	0.039	218.77	0.020	2.43	0.056	-7.63
	1:1	0.00	0.025	-0.50	0.017	-0.91	0.035	196.80	0.017	-0.91	0.050	-5.90
	1:1.5	0.00	0.028	-1.41	0.016	-1.10	0.032	176.02	0.016	-1.10	0.045	-8.32
	1:2	0.00	0.031	4.22	0.015	0.53	0.030	171.62	0.015	0.53	0.043	-2.82
	2:1	0.05	0.021	1.33	0.022	1.95	0.050	58.89	0.022	1.95	0.061	-16.26
	1.5:1	0.05	0.023	3.91	0.020	-1.03	0.047	47.44	0.020	-1.03	0.056	-11.91
	1:1	0.05	0.025	3.53	0.018	1.11	0.043	30.45	0.018	1.11	0.049	-11.61
	1:1.5	0.05	0.028	-1.84	0.016	-1.02	0.040	20.35	0.016	-1.02	0.045	-11.85
	1:2	0.05	0.030	-2.91	0.016	-3.03	0.039	26.00	0.016	-3.03	0.044	-1.44
	2:1	0.10	0.022	1.25	0.023	-1.73	0.058	-3.11	0.023	-1.73	0.061	-16.76
	1.5:1	0.10	0.023	2.84	0.021	-0.30	0.054	2.19	0.021	-0.30	0.056	-5.79
	1:1	0.10	0.025	-0.37	0.019	-2.34	0.049	-5.53	0.019	-2.34	0.050	-9.81
	1:1.5	0.10	0.028	4.66	0.017	0.57	0.045	-6.00	0.017	0.57	0.046	-8.53
	1:2	0.10	0.030	-3.06	0.016	0.75	0.043	-13.86	0.016	0.75	0.043	-15.64
1200	2:1	0.00	0.018	-1.15	0.018	-0.32	0.035	198.08	0.018	-0.33	0.051	-7.95
	1.5:1	0.00	0.019	4.73	0.016	2.13	0.032	178.85	0.016	2.13	0.046	-8.51
	1:1	0.00	0.020	4.79	0.014	0.01	0.029	161.07	0.014	0.01	0.041	-5.31
	1:1.5	0.00	0.023	3.15	0.013	2.72	0.026	146.35	0.013	2.72	0.037	-5.77
	1:2	0.00	0.025	-0.94	0.012	-1.92	0.024	137.78	0.012	-1.92	0.036	-10.63
	2:1	0.05	0.018	-3.03	0.018	-6.65	0.043	29.82	0.018	-6.65	0.050	-13.95
	1.5:1	0.05	0.018	1.72	0.017	-1.08	0.040	17.47	0.017	-1.08	0.045	-14.68
	1:1	0.05	0.020	-1.40	0.015	1.26	0.037	8.30	0.015	1.26	0.041	-14.07
	1:1.5	0.05	0.023	1.20	0.013	0.61	0.034	9.14	0.013	0.61	0.037	-6.36
	1:2	0.05	0.025	-0.68	0.013	-2.00	0.033	8.41	0.013	-2.00	0.035	-4.98
	2:1	0.10	0.018	-2.29	0.018	1.85	0.049	-7.54	0.018	1.85	0.050	-12.29
	1.5:1	0.10	0.019	-1.70	0.017	4.46	0.045	-3.88	0.017	4.46	0.045	-6.39
	1:1	0.10	0.020	-1.40	0.015	1.64	0.040	-13.71	0.015	1.65	0.040	-14.49
	1:1.5	0.10	0.023	3.15	0.014	2.73	0.037	-8.10	0.014	2.73	0.037	-8.64
	1:2	0.10	0.025	-1.75	0.013	-0.07	0.035	-5.57	0.013	-0.07	0.035	-5.75

Table G. Root mean squared errors (RMSE) and biases of estimates of p_m, p_f and ρ based on EM algorithm and Zheng et al. [14], having $p_m = 0.5$ and $p_f = 0.46$.

			$\hat{p}_m$		$\hat{p}_{f1}$		$\hat{\rho}_1$		$\hat{p}_f$		$\hat{\rho}_z$	
N	r	ρ	RMSE	Bias $\times$10000	RMSE	Bias $\times$10000	RMSE	Bias $\times$10000	RMSE	Bias $\times$10000	RMSE	Bias $\times$10000
800	2:1	0.00	0.022	2.35	0.022	-1.17	0.042	236.38	0.022	-1.17	0.061	-16.71
	1.5:1	0.00	0.023	-0.78	0.020	0.32	0.039	216.06	0.020	0.32	0.056	-16.35
	1:1	0.00	0.025	2.25	0.018	-0.90	0.035	195.11	0.018	-0.90	0.050	-6.51
	1:1.5	0.00	0.028	-0.48	0.016	-0.01	0.031	173.41	0.016	-0.01	0.045	-14.18
	1:2	0.00	0.030	0.25	0.015	-2.21	0.030	166.99	0.015	-2.21	0.043	-7.58
	2:1	0.05	0.022	-0.05	0.022	1.75	0.051	61.31	0.022	1.75	0.061	-11.92
	1.5:1	0.05	0.023	-3.42	0.020	-1.61	0.047	41.17	0.020	-1.61	0.056	-17.54
	1:1	0.05	0.025	-3.23	0.018	1.88	0.043	32.10	0.018	1.88	0.050	-11.78
	1:1.5	0.05	0.028	-0.57	0.017	0.49	0.040	25.51	0.017	0.49	0.046	-8.14
	1:2	0.05	0.030	2.84	0.016	-1.17	0.039	20.48	0.016	-1.17	0.043	-7.49
	2:1	0.10	0.022	1.09	0.023	-2.06	0.058	-8.89	0.023	-2.06	0.060	-22.15
	1.5:1	0.10	0.023	-3.42	0.021	2.49	0.054	-7.89	0.021	2.49	0.056	-16.83
	1:1	0.10	0.025	2.50	0.019	6.62	0.049	-10.28	0.019	6.62	0.050	-14.38
	1:1.5	0.10	0.028	2.85	0.017	-1.36	0.045	-11.63	0.017	-1.36	0.045	-14.05
	1:2	0.10	0.031	-4.31	0.016	1.23	0.043	-2.53	0.016	1.23	0.043	-4.10
1200	2:1	0.00	0.018	-3.97	0.018	-0.63	0.035	195.32	0.018	-0.63	0.050	-5.83
	1.5:1	0.00	0.018	0.13	0.016	1.85	0.032	177.19	0.016	1.85	0.046	-11.37
	1:1	0.00	0.020	0.75	0.014	-0.61	0.029	160.15	0.014	-0.61	0.041	-5.67
	1:1.5	0.00	0.023	-2.73	0.013	1.23	0.027	149.70	0.013	1.23	0.038	-0.92
	1:2	0.00	0.025	3.44	0.012	0.90	0.025	141.65	0.012	0.90	0.035	-0.51
	2:1	0.05	0.018	0.78	0.018	0.87	0.044	40.05	0.018	0.88	0.050	-2.50
	1.5:1	0.05	0.019	-0.46	0.017	0.21	0.040	17.31	0.017	0.21	0.046	-15.70
	1:1	0.05	0.020	-1.67	0.015	0.36	0.037	15.44	0.015	0.36	0.041	-7.16
	1:1.5	0.05	0.023	-1.82	0.013	-0.02	0.034	14.66	0.013	-0.02	0.037	-1.31
	1:2	0.05	0.025	-1.43	0.013	-0.49	0.033	8.50	0.013	-0.49	0.036	-4.67
	2:1	0.10	0.018	-0.14	0.019	3.46	0.048	-4.74	0.019	3.46	0.049	-8.77
	1.5:1	0.10	0.019	1.32	0.017	2.17	0.045	-14.01	0.017	2.17	0.045	-16.24
	1:1	0.10	0.020	-1.67	0.015	0.22	0.040	-6.53	0.015	0.22	0.041	-7.55
	1:1.5	0.10	0.023	-2.73	0.014	0.60	0.037	-1.51	0.014	0.60	0.037	-1.97
	1:2	0.10	0.025	-1.83	0.013	-1.66	0.035	-4.09	0.013	-1.66	0.035	-4.43

Table H. Root mean squared errors (RMSE) and biases of estimates of p and ρ under $H_{01} : p_m = p_f = p$, based on EM algorithm and Zheng et al. [14], having $p = 0.5$.

			$\hat{p}_{01}$		$\hat{\rho}_{01}$		$\hat{p}$		$\hat{\rho}_z$	
				Bias		Bias		Bias		Bias
N	r	ρ	RMSE	×10000	RMSE	×10000	RMSE	×10000	RMSE	×10000
800	2:1	0.00	0.016	1.89	0.042	237.27	0.016	1.93	0.061	-21.35
	1.5:1	0.00	0.015	-1.20	0.039	220.47	0.015	-1.16	0.056	-13.38
	1:1	0.00	0.015	-1.25	0.035	197.87	0.015	-1.27	0.050	-11.45
	1:1.5	0.00	0.014	-1.26	0.032	179.88	0.014	-1.30	0.045	-5.30
	1:2	0.00	0.014	-0.19	0.030	171.78	0.014	-0.21	0.044	-5.87
	2:1	0.05	0.015	-0.85	0.051	62.95	0.015	-0.82	0.061	-17.82
	1.5:1	0.05	0.015	0.56	0.047	48.94	0.015	0.55	0.056	-18.61
	1:1	0.05	0.015	0.00	0.043	32.57	0.015	0.01	0.050	-15.63
	1:1.5	0.05	0.014	0.79	0.040	19.03	0.014	0.78	0.046	-16.32
	1:2	0.05	0.014	-0.68	0.039	17.70	0.014	-0.71	0.043	-11.31
	2:1	0.10	0.016	-1.52	0.058	6.20	0.016	-1.38	0.061	-18.27
	1.5:1	0.10	0.015	-0.19	0.054	-4.67	0.015	-0.10	0.056	-20.38
	1:1	0.10	0.015	-0.39	0.048	-6.70	0.015	-0.32	0.049	-15.66
	1:1.5	0.10	0.014	1.50	0.045	-6.03	0.014	1.50	0.046	-10.94
	1:2	0.10	0.014	-0.56	0.043	-0.17	0.014	-0.45	0.043	-3.63
1200	2:1	0.00	0.012	0.89	0.035	198.31	0.012	0.90	0.050	-9.27
	1.5:1	0.00	0.012	0.92	0.033	182.69	0.012	0.92	0.046	-8.87
	1:1	0.00	0.012	-0.70	0.029	162.33	0.012	-0.71	0.041	-3.15
	1:1.5	0.00	0.011	1.45	0.026	147.13	0.011	1.42	0.038	-6.26
	1:2	0.00	0.011	0.40	0.025	145.27	0.011	0.39	0.036	5.96
	2:1	0.05	0.012	-0.59	0.043	28.60	0.012	-0.69	0.050	-22.44
	1.5:1	0.05	0.012	-0.42	0.041	33.13	0.012	-0.44	0.046	-2.29
	1:1	0.05	0.012	0.98	0.037	9.52	0.012	0.93	0.041	-17.41
	1:1.5	0.05	0.012	-0.99	0.034	10.51	0.012	-0.96	0.037	-6.86
	1:2	0.05	0.011	0.33	0.033	4.75	0.011	0.33	0.036	-9.70
	2:1	0.10	0.013	-0.39	0.049	-4.61	0.013	-0.44	0.050	-15.18
	1.5:1	0.10	0.013	0.16	0.045	-7.99	0.013	0.18	0.046	-15.53
	1:1	0.10	0.012	0.61	0.040	-12.07	0.012	0.54	0.041	-16.00
	1:1.5	0.10	0.012	1.38	0.037	-7.47	0.012	1.29	0.037	-9.70
	1:2	0.10	0.012	0.59	0.035	-7.99	0.012	0.56	0.035	-9.51

Table I. Root mean squared errors (RMSE) and biases of estimates of p_m, p_f and ρ based on EM algorithm and Zheng et al. [14], having $p_m = 0.5$ and $p_f = 0.54$.

			$\hat{p}_m$		$\hat{p}_{f1}$		$\hat{\rho}_1$		$\hat{p}_f$		$\hat{\rho}_z$	
N	r	ρ	RMSE	Bias ×10000	RMSE	Bias ×10000	RMSE	Bias ×10000	RMSE	Bias ×10000	RMSE	Bias ×10000
800	2:1	0.00	0.022	-0.08	0.022	1.28	0.042	230.96	0.022	1.28	0.061	-22.71
	1.5:1	0.00	0.023	-2.62	0.020	1.28	0.039	216.50	0.020	1.28	0.056	-14.83
	1:1	0.00	0.025	-0.51	0.018	0.84	0.035	196.97	0.018	0.84	0.050	-7.64
	1:1.5	0.00	0.028	-5.73	0.016	1.03	0.031	169.01	0.016	1.03	0.046	-24.85
	1:2	0.00	0.030	0.86	0.015	-0.11	0.030	168.64	0.015	-0.11	0.044	-11.20
	2:1	0.05	0.022	-0.26	0.022	-0.40	0.051	53.86	0.022	-0.40	0.061	-21.45
	1.5:1	0.05	0.023	2.11	0.020	0.91	0.047	55.34	0.020	0.91	0.056	-0.69
	1:1	0.05	0.025	0.66	0.018	2.13	0.043	27.58	0.018	2.13	0.051	-19.07
	1:1.5	0.05	0.028	3.21	0.017	0.91	0.040	22.98	0.017	0.91	0.046	-11.11
	1:2	0.05	0.031	7.65	0.016	-2.55	0.038	14.57	0.016	-2.55	0.043	-13.21
	2:1	0.10	0.022	-1.73	0.023	-1.73	0.058	-5.88	0.023	-1.73	0.061	-19.79
	1.5:1	0.10	0.023	2.45	0.021	-0.09	0.054	-6.82	0.021	-0.09	0.056	-15.71
	1:1	0.10	0.025	-1.50	0.019	-4.12	0.049	-10.45	0.019	-4.12	0.050	-14.41
	1:1.5	0.10	0.028	-6.04	0.017	2.42	0.044	-8.90	0.017	2.42	0.045	-11.27
	1:2	0.10	0.031	-3.36	0.016	0.65	0.042	-14.24	0.016	0.65	0.043	-16.22
1200	2:1	0.00	0.018	-0.40	0.018	0.70	0.035	195.85	0.018	0.71	0.050	-10.95
	1.5:1	0.00	0.019	2.17	0.016	-0.75	0.032	177.91	0.016	-0.75	0.046	-8.42
	1:1	0.00	0.020	-2.25	0.014	0.15	0.029	162.31	0.014	0.15	0.041	-4.64
	1:1.5	0.00	0.023	-1.92	0.013	0.63	0.026	143.49	0.013	0.63	0.037	-9.98
	1:2	0.00	0.025	-1.52	0.012	1.13	0.025	137.36	0.012	1.13	0.035	-5.74
	2:1	0.05	0.018	0.33	0.018	-1.90	0.043	25.28	0.018	-1.90	0.050	-19.78
	1.5:1	0.05	0.018	-0.03	0.016	0.79	0.040	24.33	0.016	0.79	0.046	-8.28
	1:1	0.05	0.021	0.30	0.015	-1.99	0.037	17.90	0.015	-1.99	0.041	-4.56
	1:1.5	0.05	0.023	1.65	0.013	0.53	0.034	9.78	0.013	0.53	0.038	-8.07
	1:2	0.05	0.025	-1.57	0.013	0.38	0.033	7.15	0.013	0.38	0.036	-6.43
	2:1	0.10	0.018	2.03	0.019	0.07	0.050	-7.23	0.019	0.07	0.051	-11.60
	1.5:1	0.10	0.019	4.38	0.017	-0.38	0.045	-8.46	0.017	-0.38	0.046	-11.16
	1:1	0.10	0.021	0.30	0.015	-2.03	0.040	-4.81	0.015	-2.03	0.040	-5.76
	1:1.5	0.10	0.023	-1.92	0.014	0.54	0.037	-7.76	0.014	0.54	0.037	-8.16
	1:2	0.10	0.025	-0.37	0.013	1.72	0.035	-1.89	0.013	1.72	0.035	-2.14

Table J. **Root mean squared errors (RMSE) and biases of estimates of p_m, p_f and ρ based on EM algorithm and Zheng et al. [14], having $p_m = 0.5$ and $p_f = 0.55$.**

			$\hat{p}_m$		$\hat{p}_{f1}$		$\hat{\rho}_1$		$\hat{p}_f$		$\hat{\rho}_z$	
N	r	ρ	RMSE	Bias ×10000	RMSE	Bias ×10000	RMSE	Bias ×10000	RMSE	Bias ×10000	RMSE	Bias ×10000
800	2:1	0.00	0.022	-0.02	0.021	3.20	0.043	239.53	0.021	3.20	0.062	-15.68
	1.5:1	0.00	0.023	0.01	0.020	3.32	0.038	211.95	0.020	3.32	0.056	-24.11
	1:1	0.00	0.025	1.55	0.017	1.28	0.035	194.83	0.017	1.28	0.050	-9.84
	1:1.5	0.00	0.028	0.95	0.016	-1.37	0.032	180.13	0.016	-1.37	0.046	-7.04
	1:2	0.00	0.031	11.31	0.015	-1.71	0.030	165.53	0.015	-1.71	0.043	-12.20
	2:1	0.05	0.022	-0.89	0.022	0.90	0.050	55.94	0.022	0.90	0.061	-18.51
	1.5:1	0.05	0.023	4.64	0.020	1.60	0.047	36.82	0.020	1.60	0.056	-23.55
	1:1	0.05	0.025	0.10	0.018	1.11	0.044	32.67	0.018	1.11	0.051	-12.67
	1:1.5	0.05	0.028	4.52	0.016	-1.40	0.040	25.15	0.016	-1.40	0.046	-9.07
	1:2	0.05	0.030	-0.33	0.016	-3.47	0.039	17.43	0.016	-3.47	0.043	-11.02
	2:1	0.10	0.022	-0.36	0.022	-4.10	0.058	6.04	0.022	-4.10	0.061	-7.33
	1.5:1	0.10	0.023	-0.37	0.021	2.01	0.053	-7.75	0.021	2.01	0.055	-16.48
	1:1	0.10	0.025	-0.19	0.018	-0.87	0.050	-1.19	0.018	-0.87	0.051	-5.53
	1:1.5	0.10	0.028	2.04	0.017	-1.15	0.044	-4.75	0.017	-1.15	0.045	-7.09
	1:2	0.10	0.031	-1.46	0.016	-0.12	0.043	-2.62	0.016	-0.12	0.043	-4.38
1200	2:1	0.00	0.018	-4.04	0.018	1.17	0.035	194.80	0.018	1.17	0.050	-9.69
	1.5:1	0.00	0.019	-0.30	0.016	-0.55	0.031	174.00	0.016	-0.55	0.045	-14.98
	1:1	0.00	0.021	2.73	0.014	-0.67	0.029	160.82	0.014	-0.67	0.041	-3.57
	1:1.5	0.00	0.023	1.17	0.013	-1.31	0.026	145.22	0.013	-1.31	0.038	-10.16
	1:2	0.00	0.025	-1.67	0.012	-0.04	0.025	139.60	0.012	-0.04	0.036	-6.90
	2:1	0.05	0.018	-0.96	0.018	3.09	0.043	25.82	0.018	3.09	0.050	-17.47
	1.5:1	0.05	0.018	0.38	0.016	1.51	0.041	34.23	0.016	1.51	0.046	1.27
	1:1	0.05	0.020	2.36	0.015	1.57	0.038	12.23	0.015	1.57	0.042	-10.73
	1:1.5	0.05	0.023	-2.22	0.013	-1.96	0.034	11.05	0.013	-1.96	0.037	-4.54
	1:2	0.05	0.025	3.92	0.013	0.63	0.033	8.12	0.013	0.63	0.035	-5.19
	2:1	0.10	0.018	1.40	0.019	0.32	0.049	-7.24	0.019	0.32	0.050	-13.05
	1.5:1	0.10	0.019	1.19	0.017	1.30	0.045	-4.04	0.017	1.30	0.045	-6.07
	1:1	0.10	0.020	2.36	0.015	1.67	0.041	-11.08	0.015	1.67	0.041	-12.18
	1:1.5	0.10	0.023	1.17	0.014	-1.31	0.037	-11.07	0.014	-1.31	0.037	-11.53
	1:2	0.10	0.025	-4.90	0.013	1.81	0.036	-7.57	0.013	1.81	0.036	-7.76

Table K. LRT_0, LRT_{0b} and Z_0 results of application to rheumatoid arthritis data.

A. Contingency table showing LRT_{0b} and Z_0 results at 5% level.

	$P_{Z_0} < 0.05$	$P_{Z_0} \geq 0.05$	Total
$P_{\text{LRT}_{0b}} < 0.05$	11	6	17
$P_{\text{LRT}_{0b}} \geq 0.05$	4	272	276
Total	15	278	293

B. P-values of LRT_0, LRT_{0b} and Z_0 at the 6 SNPs for $P_{\text{LRT}_{0b}} < 0.05$ and $P_{Z_0} > 0.05$.

SNP name	P_{LRT_0}	$P_{\text{LRT}_{0b}}$	P_{Z_0}
rs927074	0.055	0.029	0.066
rs1372262	0.071	0.047	0.070
rs241856	0.072	0.040	0.068
rs1883411	0.074	0.040	0.072
rs1566588	0.065	0.030	0.057
rs222398	0.066	0.043	0.063

C. P-values of LRT_0, LRT_{0b} and Z_0 at the 4 SNPs for $P_{\text{LRT}_{0b}} > 0.05$ and $P_{Z_0} < 0.05$.

SNP name	P_{LRT_0}	$P_{\text{LRT}_{0b}}$	P_{Z_0}
rs1375089	0.871	0.722	0.010
rs1402076	0.997	0.970	0.041
rs714597	0.127	0.074	0.024
rs7881297	0.127	0.154	0.033

Table L. LRT_1 and Z_1 results of application to rheumatoid arthritis data.

A. Contingency table showing LRT_1 and Z_1 results at 5% level.

	$P_{Z_1} < 0.05$	$P_{Z_1} \geq 0.05$	Total
$P_{\mathrm{LRT}_1} < 0.05$	9	1	10
$P_{\mathrm{LRT}_1} \geq 0.05$	4	279	283
Total	13	280	293

B. P-values of LRT_1 and Z_1 at the 1 SNP for $P_{\mathrm{LRT}_1} < 0.05$ and $P_{Z_1} > 0.05$.

SNP name	P_{LRT_1}	P_{Z_1}
rs2024917	0.043	0.052

C. P-values of LRT_1 and Z_1 at the 4 SNPs for $P_{\mathrm{LRT}_1} > 0.05$ and $P_{Z_1} < 0.05$.

SNP name	P_{LRT_1}	P_{Z_1}
rs1536163	0.052	0.042
rs929590	0.056	0.039
rs7881297	0.067	0.017
rs2281277	0.070	0.044

Table M. $\mathbf{LRT_2}$, $\mathbf{LRT_{2b}}$ and Z_2 results of application to rheumatoid arthritis data.

A. Contingency table showing $\mathbf{LRT_{2b}}$ and Z_2 results at 5% level.

	$P_{Z_2} < 0.05$	$P_{Z_2} \geq 0.05$	**Total**
$P_{\mathrm{LRT}_{2b}} < 0.05$	14	12	26
$P_{\mathrm{LRT}_{2b}} \geq 0.05$	2	265	267
**Total**	16	277	293

B. P-values of $\mathbf{LRT_2}$, $\mathbf{LRT_{2b}}$ and Z_2 at the 12 SNPs for $P_{\mathrm{LRT}_{2b}} < 0.05$ and $P_{Z_2} > 0.05$.

**SNP name**	P_{LRT_2}	$P_{\mathrm{LRT}_{2b}}$	P_{Z_2}
rs2001110	0.060	0.025	0.056
rs931240	0.076	0.029	0.063
rs1885445	0.066	0.038	0.061
rs1866933	0.064	0.026	0.058
rs829290	0.054	0.022	0.051
rs968859	0.090	0.035	0.085
rs981780	0.093	0.048	0.087
rs1522889	0.110	0.045	0.104
rs916208	0.093	0.043	0.087
rs1005488	0.081	0.039	0.071
rs2179978	0.091	0.038	0.066
rs764908	0.090	0.036	0.083

C. P-values of $\mathbf{LRT_2}$, $\mathbf{LRT_{2b}}$ and Z_2 at the 2 SNPs for $P_{\mathrm{LRT}_{2b}} > 0.05$ and $P_{Z_2} < 0.05$.

**SNP name**	P_{LRT_2}	$P_{\mathrm{LRT}_{2b}}$	P_{Z_2}
rs1375089	1.000	0.944	0.003
rs1402076	1.000	0.525	0.012

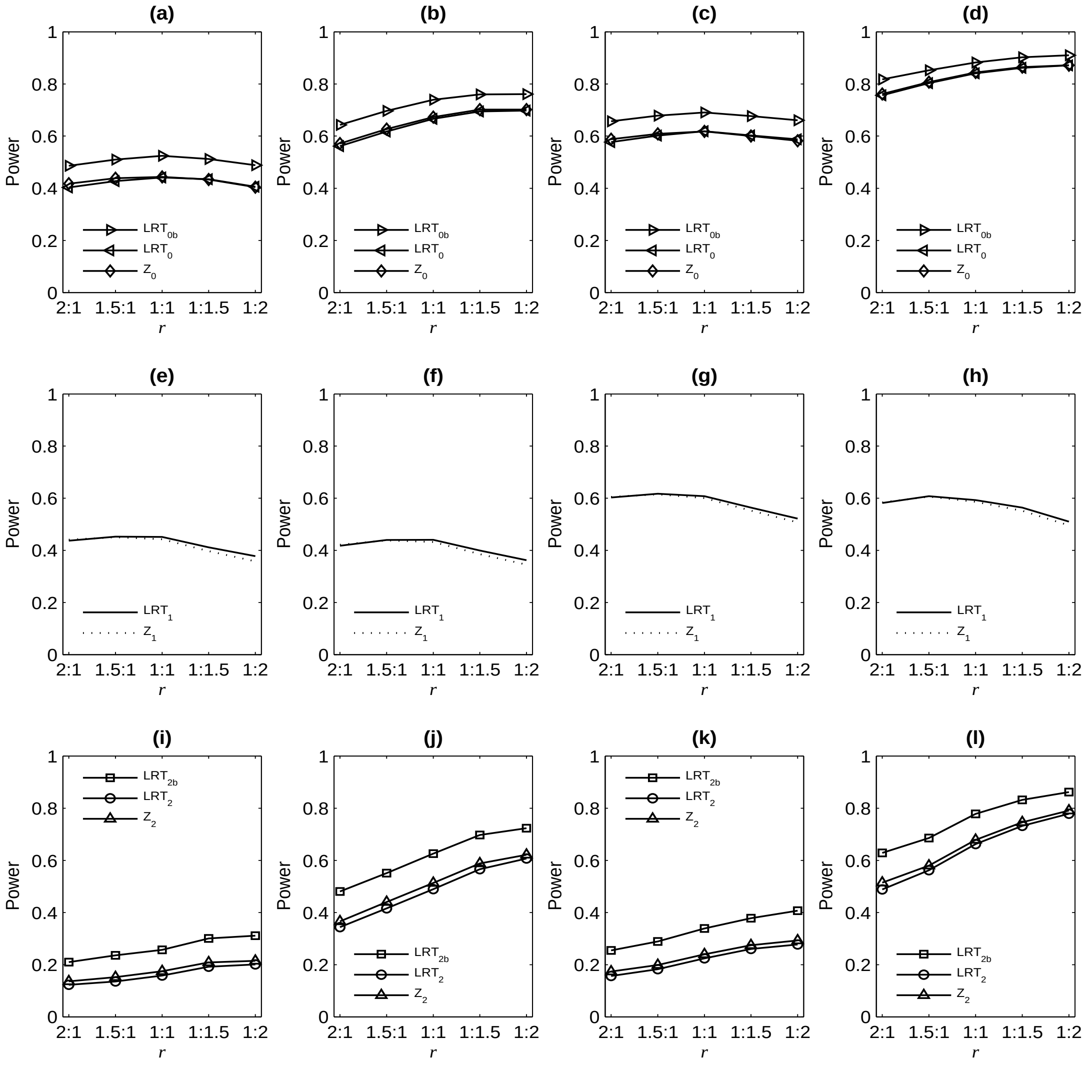

Fig. A. Simulated powers of LRT_0, LRT_{0b}, LRT_1, LRT_2, LRT_{2b}, Z_0, Z_1 and Z_2 against $r = N_m : N_f$ under $H_1 : p_m \neq p_f$ and $\rho > 0$ based on 10000 replicates with $p_m = 0.3$ and $p_f = 0.25$. In the first column: $\rho = 0.05$ and $N = 800$; in the second column: $\rho = 0.1$ and $N = 800$; in the third column: $\rho = 0.05$ and $N = 1200$; in the fourth column: $\rho = 0.1$ and $N = 1200$. In the first row, the powers of LRT_0, LRT_{0b} and Z_0 for $H_0 : p_m = p_f$ and $\rho = 0$; in the second row, the powers of LRT_1 and Z_1 for $H_{01} : p_m = p_f$; in the third row, the powers of LRT_2, LRT_{2b} and Z_2 for $H_{02} : \rho = 0$.

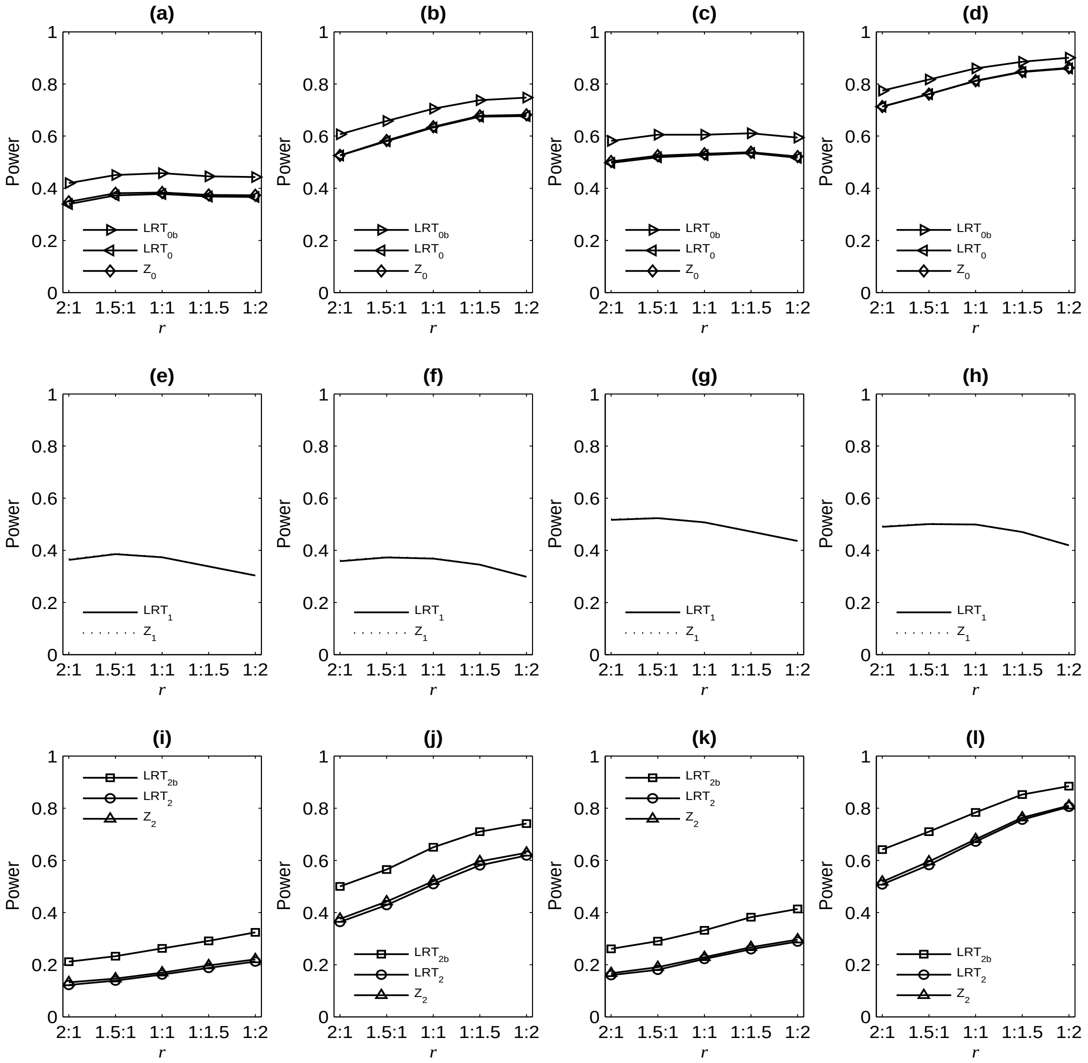

Fig. B. Simulated powers of LRT_0, LRT_{0b}, LRT_1, LRT_2, LRT_{2b}, Z_0, Z_1 and Z_2 against $r = N_m : N_f$ under $H_1 : p_m \neq p_f$ and $\rho > 0$ based on 10000 replicates with $p_m = 0.5$ and $p_f = 0.45$. In the first column: $\rho = 0.05$ and $N = 800$; in the second column: $\rho = 0.1$ and $N = 800$; in the third column: $\rho = 0.05$ and $N = 1200$; in the fourth column: $\rho = 0.1$ and $N = 1200$. In the first row, the powers of LRT_0, LRT_{0b} and Z_0 for $H_0 : p_m = p_f$ and $\rho = 0$; in the second row, the powers of LRT_1 and Z_1 for $H_{01} : p_m = p_f$; in the third row, the powers of LRT_2, LRT_{2b} and Z_2 for $H_{02} : \rho = 0$.

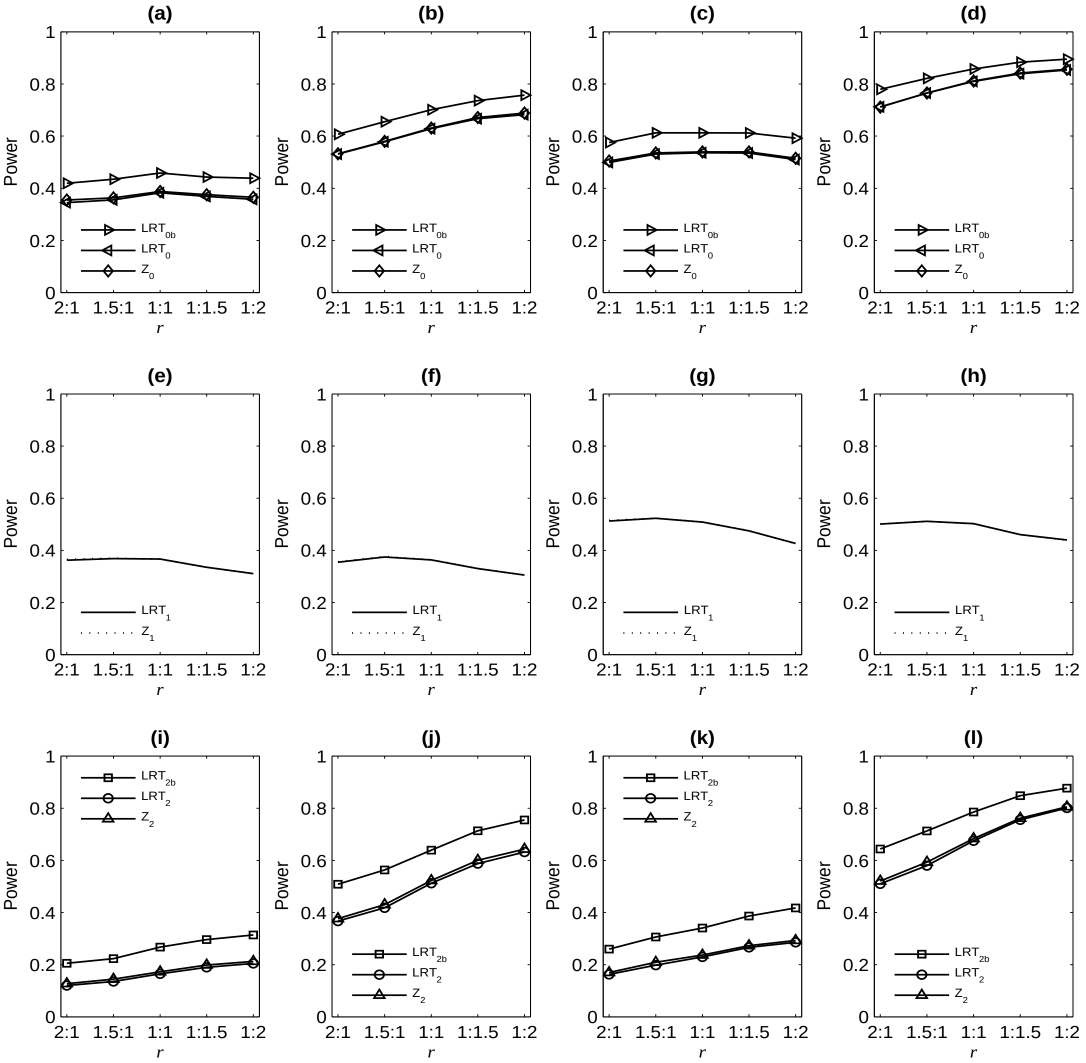

Fig. C. Simulated powers of LRT_0, LRT_{0b}, LRT_1, LRT_2, LRT_{2b}, Z_0, Z_1 and Z_2 against $r = N_m : N_f$ under $H_1 : p_m \neq p_f$ and $\rho > 0$ based on 10000 replicates with $p_m = 0.5$ and $p_f = 0.55$. In the first column: $\rho = 0.05$ and $N = 800$; in the second column: $\rho = 0.1$ and $N = 800$; in the third column: $\rho = 0.05$ and $N = 1200$; in the fourth column: $\rho = 0.1$ and $N = 1200$. In the first row, the powers of LRT_0, LRT_{0b} and Z_0 for $H_0 : p_m = p_f$ and $\rho = 0$; in the second row, the powers of LRT_1 and Z_1 for $H_{01} : p_m = p_f$; in the third row, the powers of LRT_2, LRT_{2b} and Z_2 for $H_{02} : \rho = 0$.

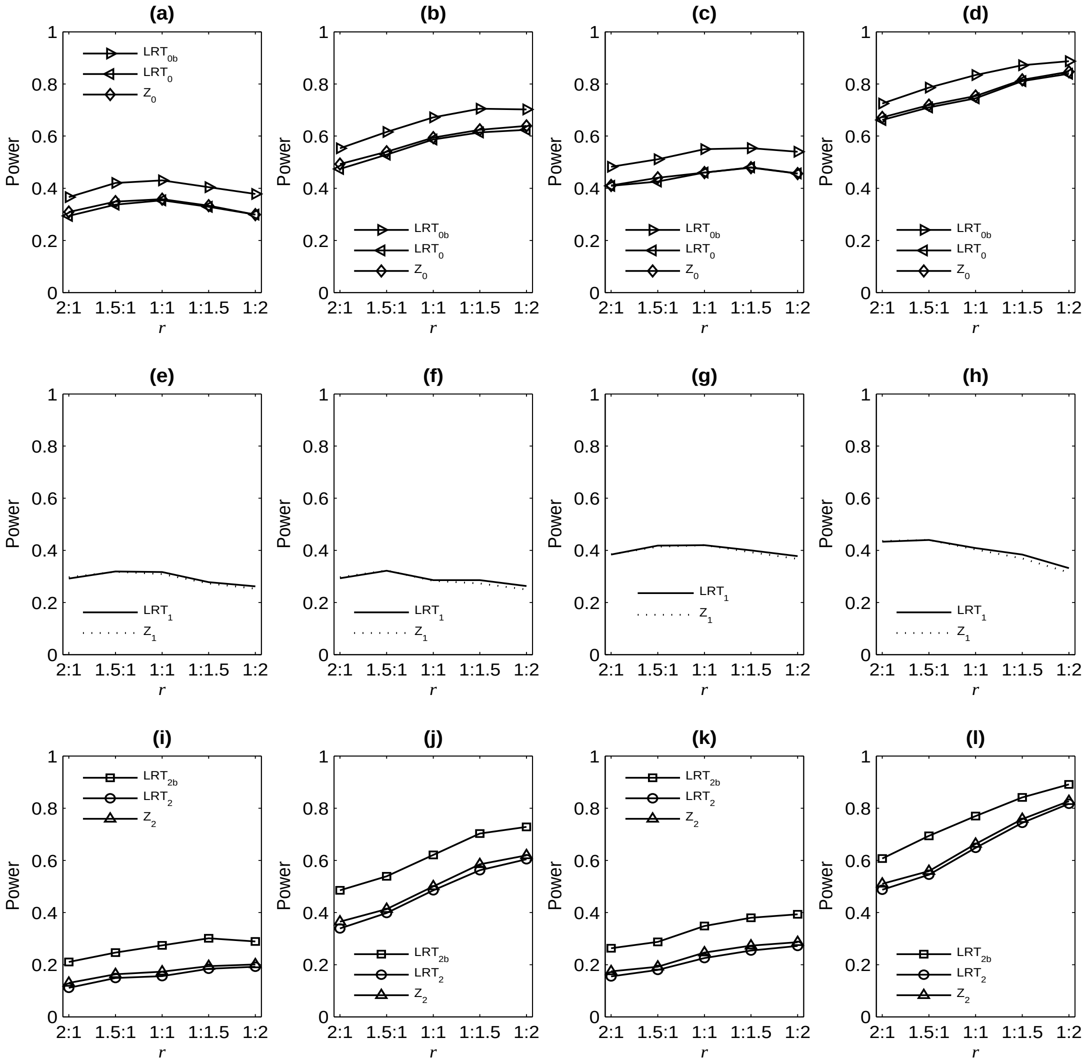

Fig. D. Simulated powers of LRT_0, LRT_{0b}, LRT_1, LRT_2, LRT_{2b}, Z_0, Z_1 and Z_2 against $r = N_m : N_f$ under $H_1 : p_m \neq p_f$ and $\rho > 0$ based on 10000 replicates with $p_m = 0.3$ and $p_f = 0.26$. In the first column: $\rho = 0.05$ and $N = 800$; in the second column: $\rho = 0.1$ and $N = 800$; in the third column: $\rho = 0.05$ and $N = 1200$; in the fourth column: $\rho = 0.1$ and $N = 1200$. In the first row, the powers of LRT_0, LRT_{0b} and Z_0 for $H_0 : p_m = p_f$ and $\rho = 0$; in the second row, the powers of LRT_1 and Z_1 for $H_{01} : p_m = p_f$; in the third row, the powers of LRT_2, LRT_{2b} and Z_2 for $H_{02} : \rho = 0$.

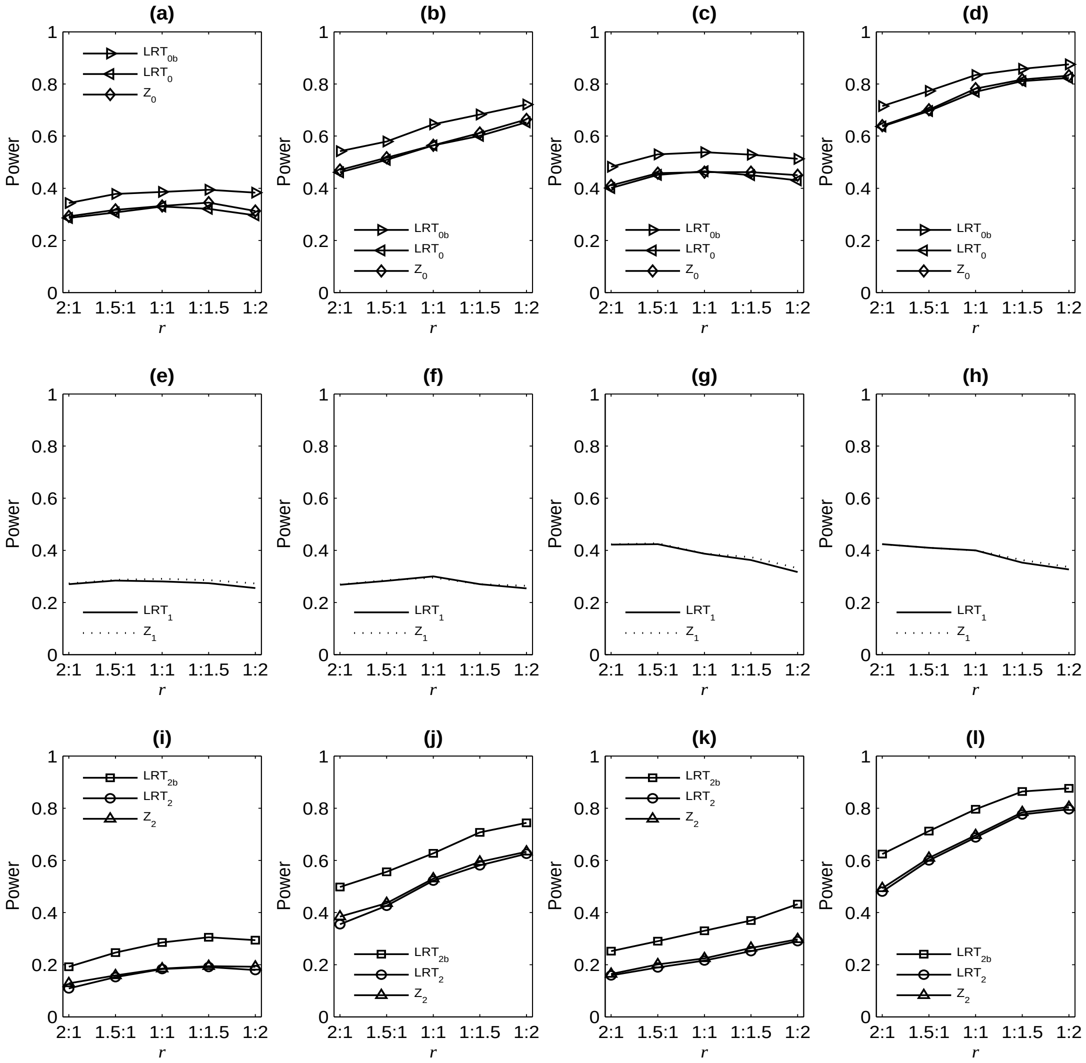

Fig. E. Simulated powers of LRT_0, LRT_{0b}, LRT_1, LRT_2, LRT_{2b}, Z_0, Z_1 and Z_2 against $r = N_m : N_f$ under $H_1 : p_m \neq p_f$ and $\rho > 0$ based on 10000 replicates with $p_m = 0.3$ and $p_f = 0.34$. In the first column: $\rho = 0.05$ and $N = 800$; in the second column: $\rho = 0.1$ and $N = 800$; in the third column: $\rho = 0.05$ and $N = 1200$; in the fourth column: $\rho = 0.1$ and $N = 1200$. In the first row, the powers of LRT_0, LRT_{0b} and Z_0 for $H_0 : p_m = p_f$ and $\rho = 0$; in the second row, the powers of LRT_1 and Z_1 for $H_{01} : p_m = p_f$; in the third row, the powers of LRT_2, LRT_{2b} and Z_2 for $H_{02} : \rho = 0$.

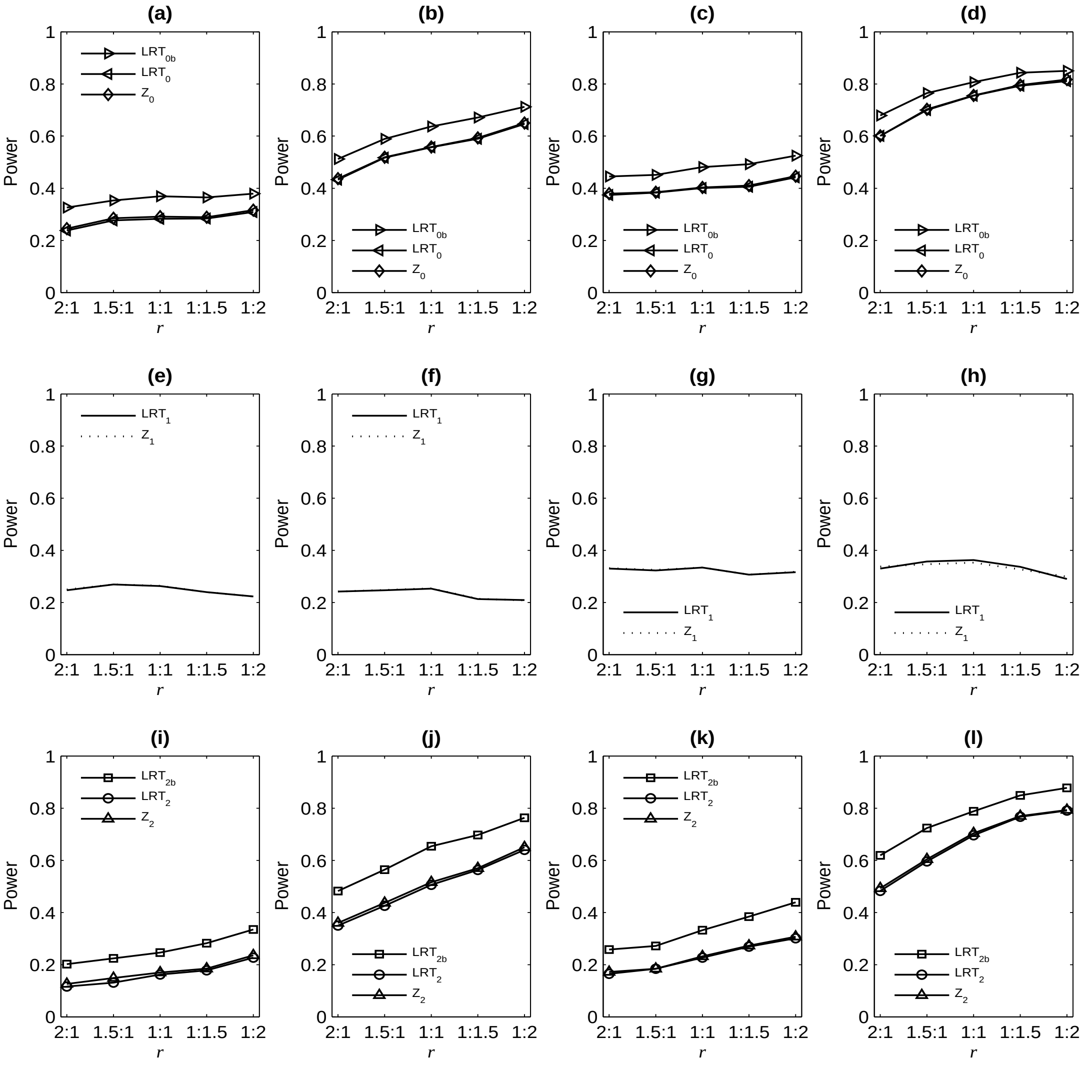

Fig. F. Simulated powers of LRT_0, LRT_{0b}, LRT_1, LRT_2, LRT_{2b}, Z_0, Z_1 and Z_2 against $r = N_m : N_f$ under $H_1 : p_m \neq p_f$ and $\rho > 0$ based on 10000 replicates with $p_m = 0.5$ and $p_f = 0.46$. In the first column: $\rho = 0.05$ and $N = 800$; in the second column: $\rho = 0.1$ and $N = 800$; in the third column: $\rho = 0.05$ and $N = 1200$; in the fourth column: $\rho = 0.1$ and $N = 1200$. In the first row, the powers of LRT_0, LRT_{0b} and Z_0 for $H_0 : p_m = p_f$ and $\rho = 0$; in the second row, the powers of LRT_1 and Z_1 for $H_{01} : p_m = p_f$; in the third row, the powers of LRT_2, LRT_{2b} and Z_2 for $H_{02} : \rho = 0$.

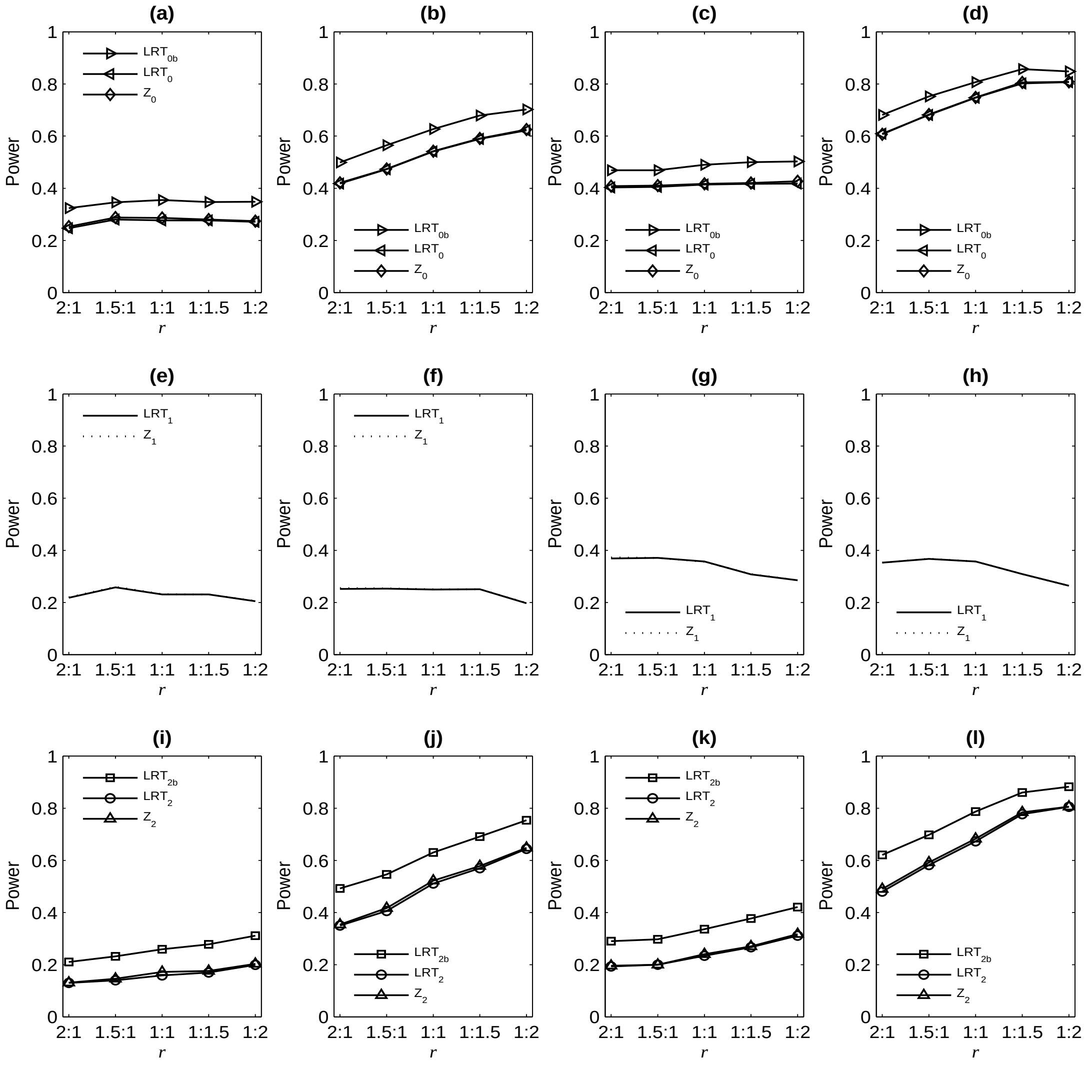

Fig. G. Simulated powers of LRT_0, LRT_{0b}, LRT_1, LRT_2, LRT_{2b}, Z_0, Z_1 and Z_2 against $r = N_m : N_f$ under $H_1 : p_m \neq p_f$ and $\rho > 0$ based on 10000 replicates with $p_m = 0.5$ and $p_f = 0.54$. In the first column: $\rho = 0.05$ and $N = 800$; in the second column: $\rho = 0.1$ and $N = 800$; in the third column: $\rho = 0.05$ and $N = 1200$; in the fourth column: $\rho = 0.1$ and $N = 1200$. In the first row, the powers of LRT_0, LRT_{0b} and Z_0 for $H_0 : p_m = p_f$ and $\rho = 0$; in the second row, the powers of LRT_1 and Z_1 for $H_{01} : p_m = p_f$; in the third row, the powers of LRT_2, LRT_{2b} and Z_2 for $H_{02} : \rho = 0$.

Simulated size/powers of $\mathbf{LRT_0}$, $\mathbf{LRT_{0b}}$, $\mathbf{LRT_1}$, $\mathbf{LRT_2}$, $\mathbf{LRT_{2b}}$, $\boldsymbol{Z_0}$, $\boldsymbol{Z_1}$ and $\boldsymbol{Z_2}$ under $\boldsymbol{p_m = p_f}$ and $\boldsymbol{\rho > 0}$

Fig. H plots the simulated size/powers of the methods proposed in this article and the existing methods against $r = N_m : N_f$ under $H_{01} : p_m = p_f = p$ with $p = 0.3$ for different values of inbreeding coefficient ($\rho = 0.05$ and 0.1) and different sample sizes ($N = 800$ and 1200). The four subplots in the second row of Fig. H give the corresponding simulated size of LRT_1 and Z_1, while the subplots in the first and third rows of the figure are the simulated powers of LRT_0, LRT_{0b} and Z_0, and LRT_2, LRT_{2b} and Z_2, respectively. From the figure, we find that LRT_1 and Z_1 control the size well. The power of LRT_{0b} is higher than both LRT_0 and Z_0, and LRT_0 and Z_0 have the similar performance in power, regardless of inbreeding coefficient or sample size. When r changes from 2:1 to 1:2 (i.e., the number of female individuals is larger and larger), the powers of LRT_0, LRT_{0b} and Z_0 increase under $p_m = p_f$. When the sample size increases, all the three test statistics are more powerful. As for the tests for $H_{02} : \rho = 0$, the power of LRT_{2b} is larger than both LRT_2 and Z_2, and LRT_2 and Z_2 almost have the similar performance in power. When the number of female individuals increases, the powers of all the three test statistics increase. When the simulation setting is fixed, LRT_2, LRT_{2b} and Z_2 with one degree of freedom are more powerful than LRT_0, LRT_{0b} and Z_0 with two degrees of freedom, respectively, because the true model is $p_m = p_f$ and $\rho > 0$. In addition, when ρ changes from 0.05 to 0.1, LRT_0, LRT_{0b}, Z_0, LRT_2, LRT_{2b} and Z_2 are more powerful. Fig. I shows the corresponding results for $p = 0.5$, which are similar to those mentioned above.

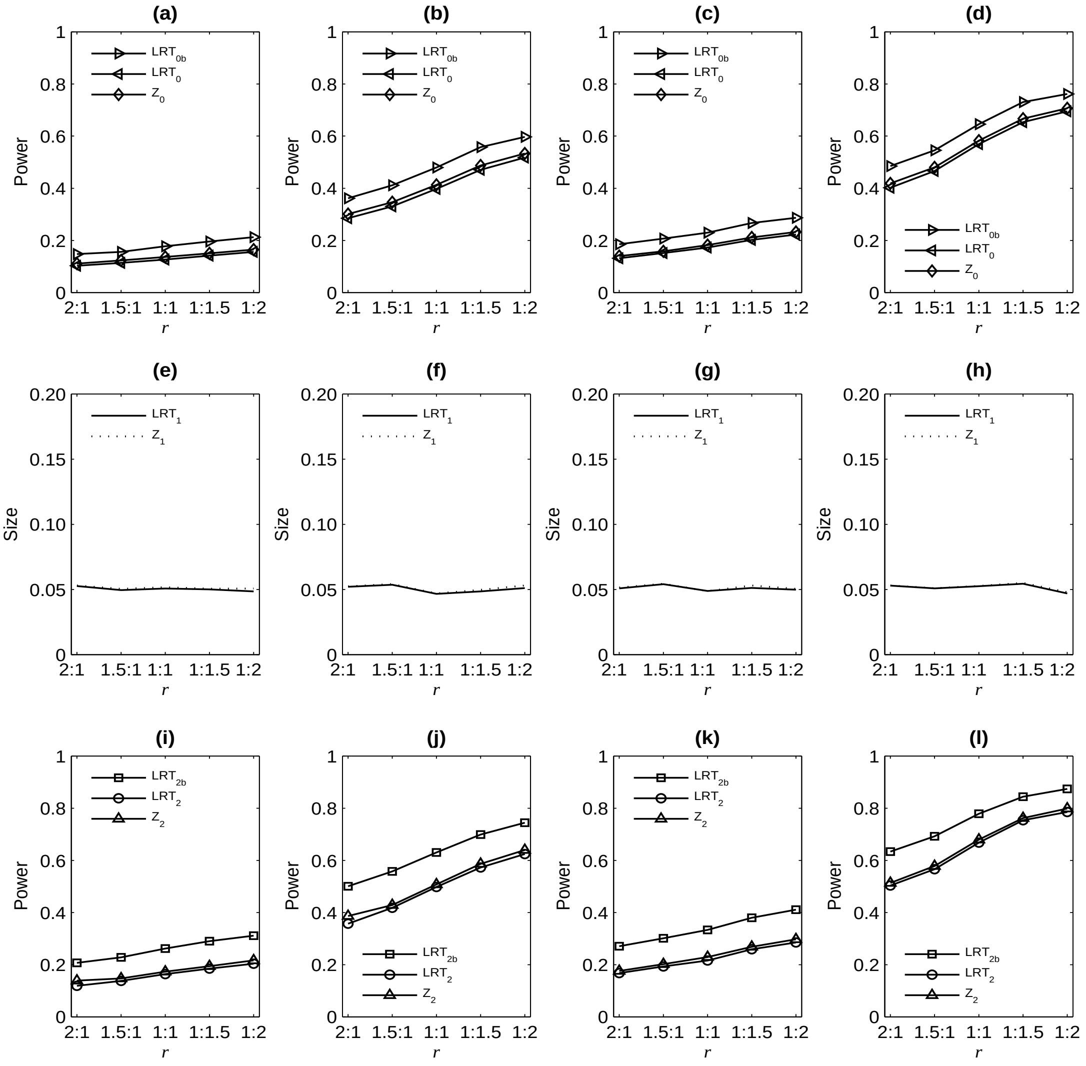

Fig. H. Simulated size/powers of LRT_0, LRT_{0b}, LRT_1, LRT_2, LRT_{2b}, Z_0, Z_1 and Z_2 against $r = N_m : N_f$ under $H_{01} : p_m = p_f = p$ based on 10000 replicates with $p = 0.3$. In the first column: $\rho = 0.05$ and $N = 800$; in the second column: $\rho = 0.1$ and $N = 800$; in the third column: $\rho = 0.05$ and $N = 1200$; in the fourth column: $\rho = 0.1$ and $N = 1200$. In the first row, the powers of LRT_0, LRT_{0b} and Z_0 for $H_0 : p_m = p_f$ and $\rho = 0$; in the second row, the size of LRT_1 and Z_1 for $H_{01} : p_m = p_f$; in the third row, the powers of LRT_2, LRT_{2b} and Z_2 for $H_{02} : \rho = 0$.

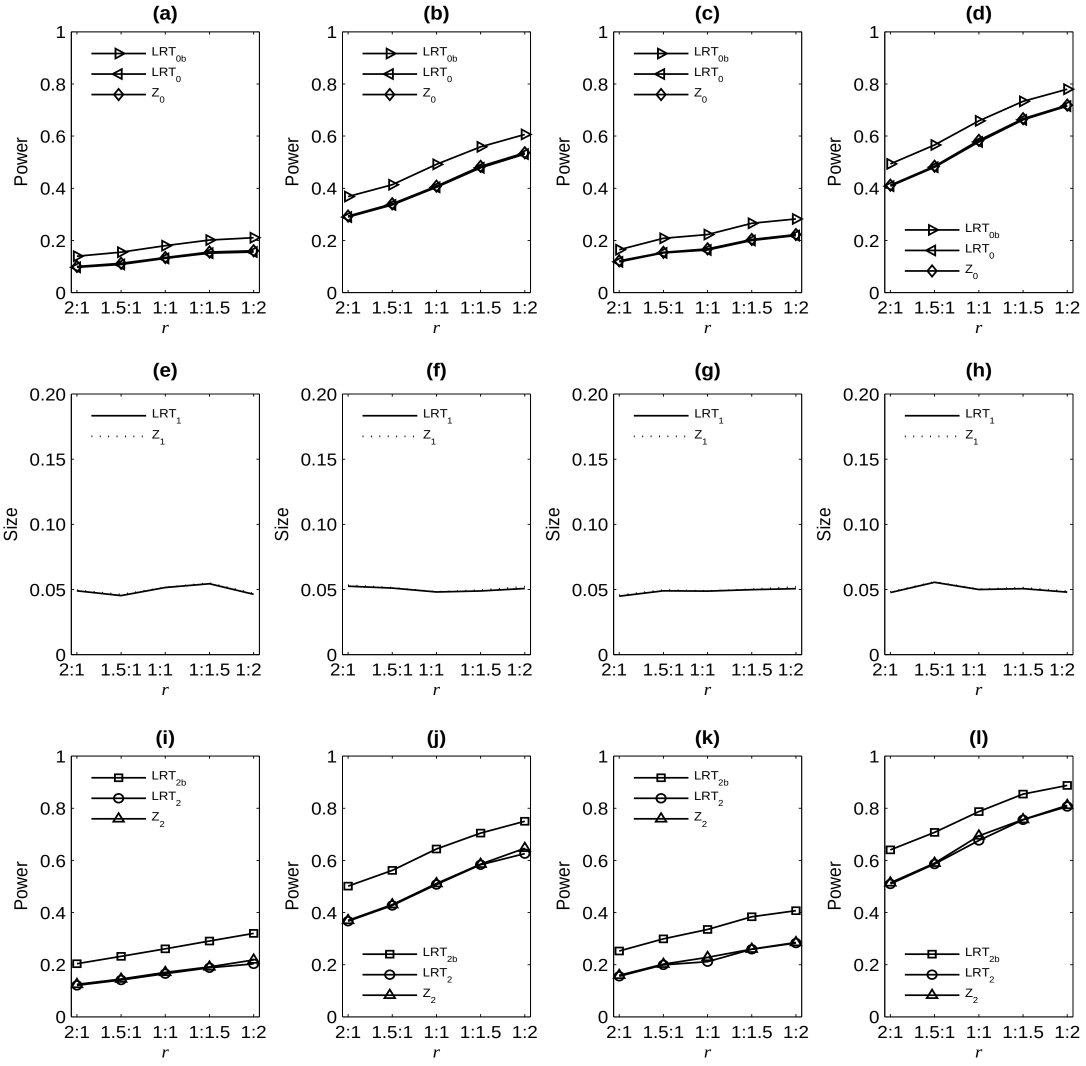

Fig. I. Simulated size/powers of LRT_0, LRT_{0b}, LRT_1, LRT_2, LRT_{2b}, Z_0, Z_1 and Z_2 against $r = N_m : N_f$ under $H_{01} : p_m = p_f = p$ based on 10000 replicates with $p = 0.5$. In the first column: $\rho = 0.05$ and $N = 800$; in the second column: $\rho = 0.1$ and $N = 800$; in the third column: $\rho = 0.05$ and $N = 1200$; in the fourth column: $\rho = 0.1$ and $N = 1200$. In the first row, the powers of LRT_0, LRT_{0b} and Z_0 for $H_0 : p_m = p_f$ and $\rho = 0$; in the second row, the size of LRT_1 and Z_1 for $H_{01} : p_m = p_f$; in the third row, the powers of LRT_2, LRT_{2b} and Z_2 for $H_{02} : \rho = 0$.

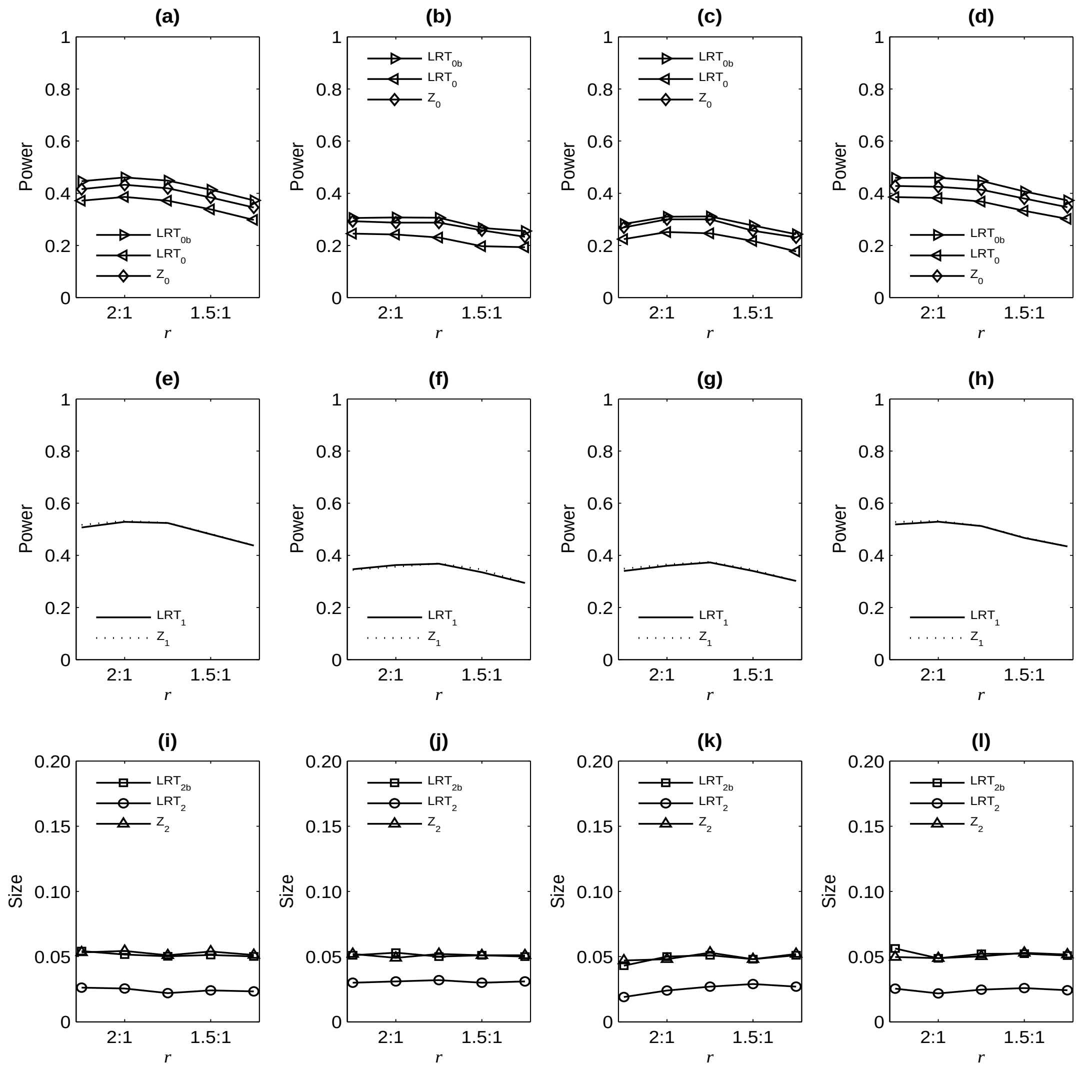

Fig. J. Simulated size/powers of LRT_0, LRT_{0b}, LRT_1, LRT_2, LRT_{2b}, Z_0, Z_1 and Z_2 against $r = N_m : N_f$ under $H_{02} : \rho = 0$ based on 10000 replicates with $p_m = 0.5$ and $N = 1200$. In the first column: $p_f = 0.45$; in the second column: $p_f = 0.46$; in the third column: $p_f = 0.54$; in the fourth column: $p_f = 0.55$. In the first row, the powers of LRT_0, LRT_{0b} and Z_0 for $H_0 : p_m = p_f$ and $\rho = 0$; in the second row, the power of LRT_1 and Z_1 for $H_{01} : p_m = p_f$; in the third row, the size of LRT_2, LRT_{2b} and Z_2 for $H_{02} : \rho = 0$.

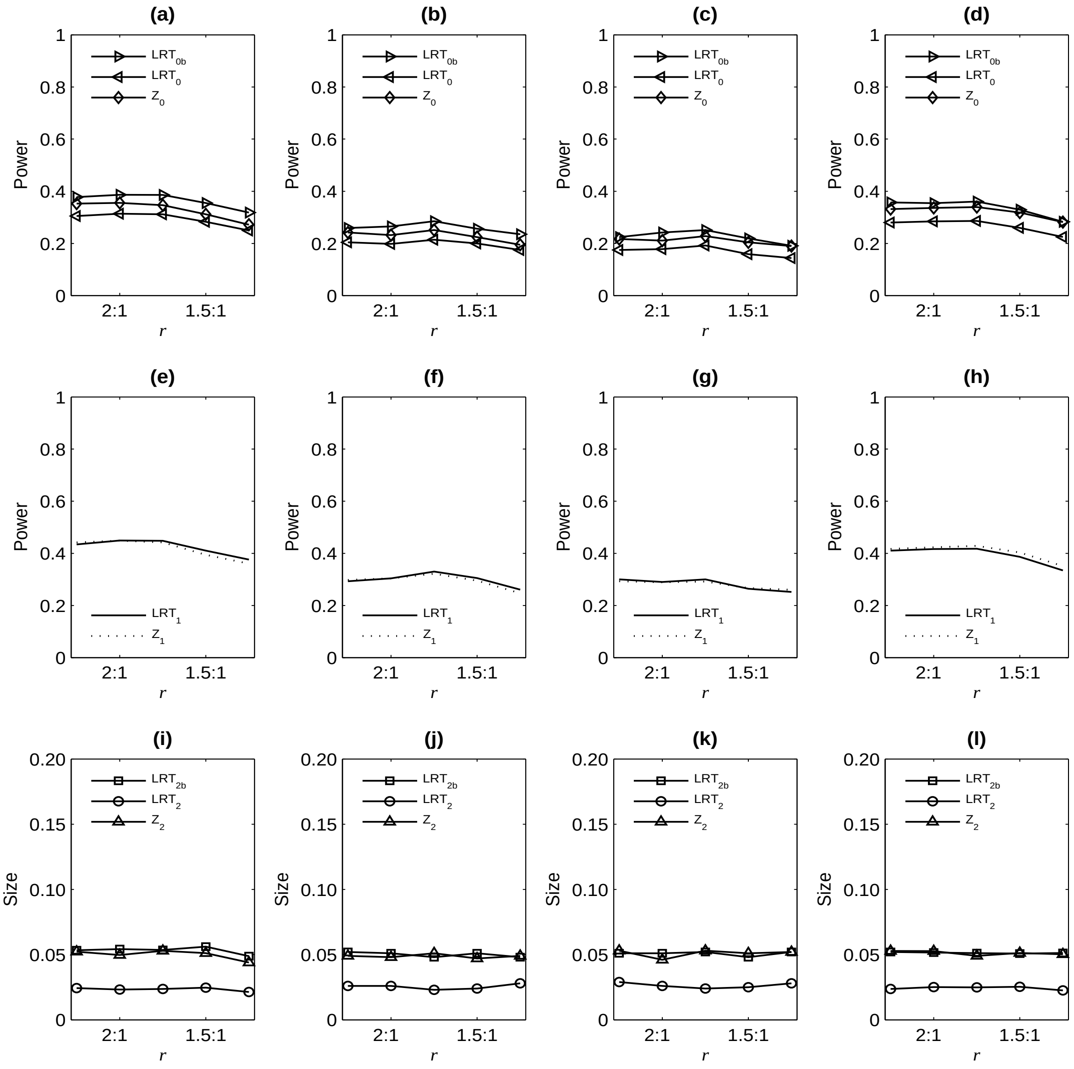

Fig. K. Simulated size/powers of LRT_0, LRT_{0b}, LRT_1, LRT_2, LRT_{2b}, Z_0, Z_1 and Z_2 against $r = N_m : N_f$ under $H_{02} : \rho = 0$ based on 10000 replicates with $p_m = 0.3$ and $N = 800$. In the first column: $p_f = 0.25$; in the second column: $p_f = 0.26$; in the third column: $p_f = 0.34$; in the fourth column: $p_f = 0.35$. In the first row, the powers of LRT_0, LRT_{0b} and Z_0 for $H_0 : p_m = p_f$ and $\rho = 0$; in the second row, the power of LRT_1 and Z_1 for $H_{01} : p_m = p_f$; in the third row, the size of LRT_2, LRT_{2b} and Z_2 for $H_{02} : \rho = 0$.

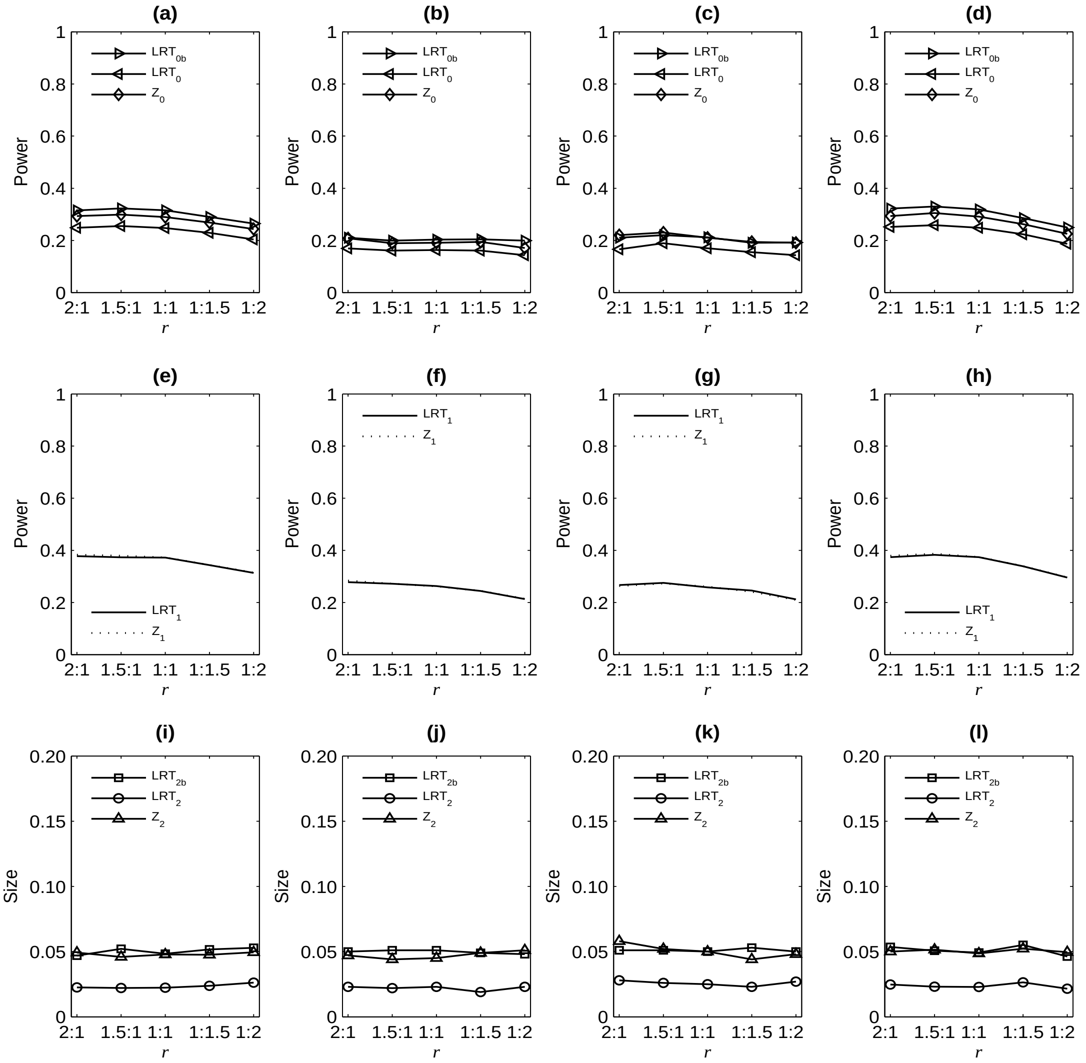

Fig. L. Simulated size/powers of LRT_0, LRT_{0b}, LRT_1, LRT_2, LRT_{2b}, Z_0, Z_1 and Z_2 against $r = N_m : N_f$ under $H_{02} : \rho = 0$ based on 10000 replicates with $p_m = 0.5$ and $N = 800$. In the first column: $p_f = 0.45$; in the second column: $p_f = 0.46$; in the third column: $p_f = 0.54$; in the fourth column: $p_f = 0.55$. In the first row, the powers of LRT_0, LRT_{0b} and Z_0 for $H_0 : p_m = p_f$ and $\rho = 0$; in the second row, the power of LRT_1 and Z_1 for $H_{01} : p_m = p_f$; in the third row, the size of LRT_2, LRT_{2b} and Z_2 for $H_{02} : \rho = 0$.
